# Melanin-Binding-Based Discovery of Topically Instilled Carbonic Anhydrase Inhibitors for Targeted Delivery and Prolonged Action in the Eye

**DOI:** 10.1021/acs.molpharmaceut.4c00694

**Published:** 2025-01-09

**Authors:** Annika Valtari, Stanislav Kalinin, Janika Jäntti, Pekka Vanhanen, Martina Hanzlikova, Arun Tonduru, Katja Stenberg, Tapani Viitala, Kati-Sisko Vellonen, Elisa Toropainen, Marika Ruponen, Arto Urtti

**Affiliations:** † School of Pharmacy, University of Eastern Finland, Yliopistonranta 1 C, 70210 Kuopio, Finland; ‡ Drug Research Program, Faculty of Pharmacy, University of Helsinki, Viikinkaari 5, 00014 Helsinki, Finland; § Pharmaceutical Sciences Laboratory, Faculty of Science and Engineering, Åbo Akademi University, Tykistökatu 6A, 20520 Turku, Finland

**Keywords:** intraocular pressure, melanin binding, carbonic anhydrase inhibitor, drug discovery, drug delivery, sustained delivery, glaucoma, eye drops

## Abstract

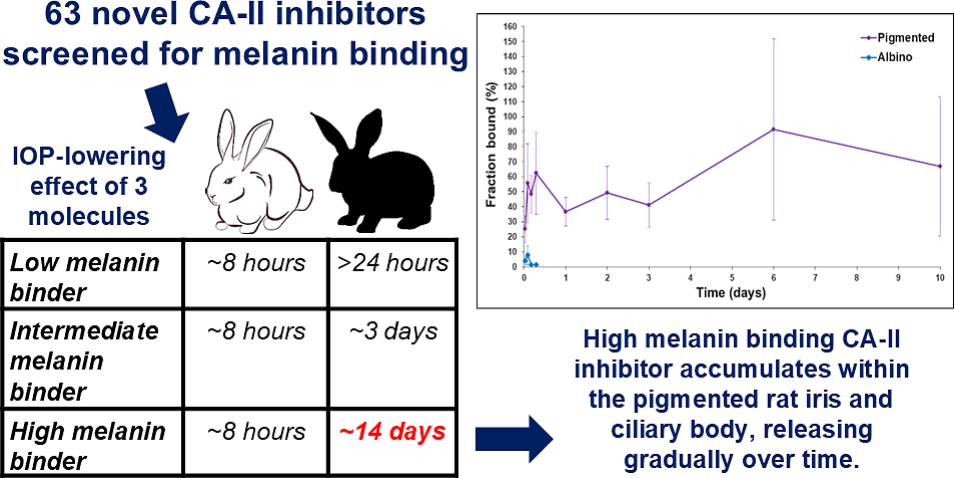

Glaucoma is a vision-threatening disease that is currently treated with intraocular-pressure-reducing eyedrops that are instilled once or multiple times daily. Unfortunately, the treatment is associated with low patient adherence and suboptimal treatment outcomes. We developed carbonic anhydrase II inhibitors (CAI-II) for a prolonged reduction of intraocular pressure (IOP). The long action is based on the melanin binding of the drugs that prolongs ocular drug retention and response. Overall, 63 new CAI-II compounds were synthesized and tested for melanin binding in vitro. Carbonic anhydrase affinity and IOP reduction of selected compounds were tested in rabbits. Prolonged reduction of IOP in pigmented rabbits was associated with increasing melanin binding of the compound. Installation of a single eye drop of a high melanin binder carbonic anhydrase inhibitor (CAI) resulted in ≈2 weeks’ decrease of IOP, whereas the effect lasted less than 8 h in albino rabbits. Duration of the IOP response correlated with melanin binding of the compounds. Ocular pharmacokinetics of a high melanin binder compound was studied after eye drop instillation to the rat eyes. The CAI showed prolonged drug retention in the pigmented iris-ciliary body but was rapidly eliminated from the albino rat eyes. The melanin-bound drug depot maintained effective free concentrations of CAI in the ciliary body for several days after application of a single eye drop. In conclusion, melanin binding is a useful tool in the discovery of long-acting ocular drugs.

## 1 Introduction

Melanin is a natural pigment that is present in high quantities in ocular tissues, such as retinal pigment epithelium, choroid, iris, and ciliary body. Melanin binding of drugs may lead to their prolonged retention and accumulation in the pigmented tissues.^1,2^ Examples include atropine,^3^ pilocarpine,^4^ timolol,^5^ and betaxolol.^6^ Furthermore, melanin binding doubled the duration of mydriatic response after an eye drop instillation.^3^ Recent rat study with 13 intravenously injected drugs showed that melanin binding increased the drug exposure (AUC) in the pigmented eyes even 100-fold compared to albino rats.^2^ Despite these observations, melanin binding has not been used as a design criterion in small molecule drug discovery. The equilibrium of bound and unbound drugs in the pigmented tissues in vivo is not understood, even though it defines how the drug accumulation to pigmented tissue affects drug response. We hypothesize that melanin binding can be utilized to develop drugs that are targeted to pigmented tissues and exert prolonged response in the eye.

The carbonic anhydrase II (CA-II) enzyme is a target for intraocular pressure (IOP) lowering carbonic anhydrase inhibitors (CAIs) for glaucoma treatment.^7^ These compounds (e.g., dorzolamide, brinzolamide, acetazolamide) decrease aqueous humor formation in the ciliary body.^8^ Unfortunately, application of topical eye drops results in low ocular bioavailability, transient drug retention, and short duration of IOP reduction. For example, the absolute ocular bioavailability of topical brinzolamide in rabbits was only 0.10%.^9^ Since CAI eye drops are instilled 2–3 times daily to control IOP, the patient adherence in relatively asymptomatic glaucoma is only ≈50%. This leads to suboptimal treatment, and even loss of vision.^10^ Improved approaches are needed for the treatment of glaucoma.

Since the ciliary epithelium is pigmented, pharmacokinetics and IOP responses of CAIs might be affected by their melanin binding. Lipophilicity, ring structures, and the basic nature of the compounds are known to correlate with their melanin binding properties.^11^ Since these features are common among our recently reported CAI-II molecules, they may have potential for targeted delivery into the pigmented tissues.^12−19^ We hypothesize that melanin binding may be a useful drug design criterion for targeted accumulation to pigmented tissues and a prolonged duration of IOP-lowering action. Therefore, we studied in vitro melanin binding of 63 new CAI-II compounds and tested selected compounds for IOP-lowering effects in albino and pigmented rabbits after eye drop instillation. Then, the ocular pharmacokinetics of the highest melanin binder was studied in albino and pigmented rats. The results show the remarkable impact of melanin binding on ocular pharmacokinetics and IOP reduction, illustrating the power of melanin binding-led optimization in ocular drug discovery.

## 2 Materials and Methods

### 2.1 In Vitro Melanin Binding Studies

Synthesis and chemical characterization of the experimental carbonic anhydrase II inhibitors have been published previously^12−19^ and the molecular structures of the compounds are presented in Appendix Table 1. The CAI-II compounds were selected for this study based on potent CA inhibition *K*_i(hCA-II)_ at subnanomolar to nanomolar concentrations^12−19^ and expected permeability in the cornea and conjunctiva (Appendix Table 2). To increase the chances of identifying a small molecule with optimal characteristics, different chemotypes, including benzenesulfonamides, 5-membered heterocyclic sulfonamides, and mono- and disubstituted sulfamides, were investigated.

Melanin binding of the CAI-II compounds was studied with a Monolith NT.115 pico (NanoTemper Technologies GmbH, Munich, Germany) device using the method of Hellinen et al. (2020).^20^ In this method, the natural fluorescence of melanin is utilized, and the affinity of the compound to melanin (i.e., dissociation constant, *K*_d_) is based on either thermophoresis (temperature induced changes in diffusion) or concentration-dependent differences in the initial fluorescence (IF) (i.e., raw fluorescence counts before thermophoresis). Water-soluble melanin nanoparticles were prepared from synthetic melanin (Sigma-Aldrich) as described earlier.^20,21^ Penicillin G, atropine, brimonidine, brinzolamide, and dorzolamide were used as reference compounds.

In the assay, the concentration of melanin nanoparticles was 0.5 mg/mL (i.e., 12.5 μM based on estimated molecular weight of 40 kDa).^20,21^ Fluorescence was measured with nanoblue detector at 60% LED light excitation power or with pico-red detector at 20% LED light excitation power (depending on the autofluorescence of melanin nanoparticles) and high-infrared laser (MST power). If ligand induced changes were observed in the fluorescence counts, the IF counts were used for *K*_d_ determination. Otherwise, normalized fluorescence signals and the ratio of relative fluorescence after heating (MST mode) were used. The MST software was used in expert mode to allow heating for 30 s. Dissociation constants (*K*_d_) were determined with Langmuir binding isotherm and built-in analysis tools of the MST instrument as described earlier.^20^ The *K*_d_ values were used to classify melanin binding to high (<65 μM), intermediate (65–650 μM), or low binders (>650 μM) and to estimate their unbound fraction in vivo (high <1%, intermediate 1–10% or low >10%) according to a previous classification.^20^

As ligand autofluorescence may interfere with the assay, their autofluorescence values were pretested with the detector used in experiment (nanoblue or pico-red). Fluorescence of the ligand should be below 20% of melanin fluorescence. Sample aggregation and adsorption to the capillaries were used also as exclusion criteria.

### 2.2 Multiparametric Surface Plasmon Resonance (MP-SPR)

Interaction kinetic studies were performed on a fully automated, multiparametric dual-wavelength (670 and 785 nm) surface plasmon resonance instrument (MP-SPR Navi 220A, BioNavis, Tampere, Finland) equipped with two parallel flow channels. All MP-SPR measurements were conducted at 25 °C using carboxymethyl dextran hydrogel-coated (CMD-3DM) sensors (BioNavis) and were based on the protocol described by Rogez-Florent et al.^22^

#### 2.2.1 Immobilization of the hCAII Isoenzyme

Human carbonic anhydrase isoenzyme II (Sigma) was immobilized using a standard amine coupling procedure with the Amine Coupling Kit (BioNavis) and phosphate buffered saline (PBS) (20 mM phosphate buffer, 150 mM NaCl, pH 7.4) as the background buffer. Before immobilization, the sensor surface was cleaned with 2 M sodium chloride and 10 mM sodium hydroxide for 6 min at a flow rate of 30 μL/min. Then, a constant flow rate of 10 μL/min was maintained throughout the immobilization steps. The surface was activated for 7 min with 200 mM 1-ethyl-3-(3-(dimethylamino)propyl)-carbodiimide (EDC) and 50 mM *N*-hydroxysuccinimide, immediately followed by a 5 min injection of 45 μg/mL hCAII solution in 10 mM acetate buffer, pH 5.5. In the reference flow channel, no enzyme was immobilized. Residual activated groups of the dextran matrix were deactivated by a 7 min injection of 1 M ethanolamine HCl, pH 8.3.

#### 2.2.2 Interaction Studies

For analyzing CAIs, 1% (v/v) DMSO in PBS, pH 7.4, was used as a running buffer. All CAIs were dissolved in 100% DMSO and stored as 50 mM stock solutions. These stock solutions were further diluted with the running buffer and analyzed using a 3-fold (3–1000 nM) dilution series. The solubility of CAIs in the running buffer was confirmed by a microplate nephelometer (Nepheloskan Ascent, Labsystems, Finland). Each analyte was injected at a flow rate of 100 μL/min, with an association time of 2 min and a dissociation time of 10 min. The extended dissociation time ensured complete dissociation of the analyte from the sensor surface, eliminating the need for an additional regeneration step. Seven concentrations were measured for each CAI per cycle, and a “wash all” step with running buffer was performed at the end of each cycle to minimize the carry over effects. Each CAI were assayed in triplicate across three independent experiments.

#### 2.2.3 Data Analysis

The acquired data were processed using MP-SPR Navi Data Viewer software (version 6.7.0.1, BioNavis) employing the weighted centroid method for determining the SPR peak angular position. Signals from the reference flow channel were subtracted from the corresponding signals of the flow channel containing immobilized hCAII. The moment of sample injection was selected as the zero-time point, where both the time and MP-SPR response were set to zero. Processed data were globally fitted to a simple 1:1 interaction model using TraceDrawer software (version 1.9.2, Ridgeview Instruments AB, Sweden) to obtain kinetic parameters.

### 2.3 Prediction of Corneal and Conjunctival Permeability

Corneal and conjunctival permeabilities of the CAI compounds brinzolamide and dorzolamide were estimated theoretically. ACDLabs 12.0 software was used to estimate the values of chemical descriptors that were then used in quantitative structure property equations to estimate corneal and conjunctiva permeability values.^23,24^

### 2.4 Animals

#### 2.4.1 IOP Study

Five 3–5 months old albino (New Zealand White, all females) and five 3–6 months old pigmented rabbits (Chinchilla Bastard, 1 male and 4 females) were purchased from Charles River Laboratories, UK and Germany, respectively. Prior to the experiments, the rabbits were habituated to handling, and the baseline values of IOP were measured. During the experiments, rabbits weighed 2.4–4.0 kg.

#### 2.4.2 Pharmacokinetic Study

Eight albino rats (RccHan/Wist) and 18 pigmented rats (HsdOla/LH) were obtained from Envigo Laboratories B.V. (The Netherlands) and were used in pharmacokinetic experiments. Additional 13 albino and 13 pigmented rats were used in generation of the analytical standards. Young adult (3–4 months; 340–445 g) rats were used.

#### 2.4.3 Housing and Feeding

All animals were housed in individual cages in a temperature- and humidity-controlled environment with 12 h dark–light cycles. The animals were fed a normal pellet diet and water ad libitum. Animal experiments were carried out at the University of Eastern Finland (license ESAVI/27769/2020) and were approved by the National Project Authorization Board (EU directive 2010/63/EU).

### 2.5 IOP Studies in Rabbits

#### 2.5.1 Preparation of Eye Drops

Compositions of CAI eye drops are listed in Table 1. First, stock solutions of CAIs (250 mg/mL) in dimethyl sulfoxide (Sigma-Aldrich, Germany) were prepared. These stock solutions were diluted with PBS (pH 7.4) to obtain 1 mg/mL (0.1%) or 10 mg/mL (1%) solutions. Water-solubility of the compounds was enhanced with 2-hydroxypropyl-β-cyclodextrin (Sigma-Aldrich, Germany) (Table 1).

**Table 1:** Composition of CAI Solutions That Were Used as Eye Drops in IOP Studies in Rabbits

solution (%)	CAI (mg/mL)	dimethyl sulfoxide (% v/v)	2-hydroxypropyl-β-cyclodextrin (mg/mL)
A01 0.1	1	0.4	10
**A01 1.0**	10	4	100
A12 0.1	1	0.4	
**A12 0.5**	5	2	100
A22 0.1	1	0.4	
**A22 0.5**	5	2	100

The CAI solutions were tested in albino rabbits (eye drops of 0.1, and 0.5 or 1.0%) and in pigmented rabbits (0.5 or 1.0%). The novel CAI concentrations in solutions were determined based on the drug solubility with the aim to load the highest possible drug concentration to the single eye drop. Dorzolamide hydrochloride 20 mg/mL eye drops (Trusopt, Santen Ltd.) were used as the positive control, and PBS was used as the negative control.

#### 2.5.2 Ocular Hypertension in Pigmented Rabbits

Chinchilla Bastard rabbits had a too low normal ocular pressure so that no IOP reduction was obtained with dorzolamide (positive control). Baseline IOP of pigmented rabbits was raised with two intravitreal triamcinolone acetonide injections (Triesence 40 mg/mL, Novartis Finland Ltd.) with 1 week interval to each eye. This method^25,26^ may induce ocular hypertension even up to 60 days in rabbits.^25^ With this approach, the pigmented and New Zealand White rabbits had the same baseline IOP levels.

The rabbits were anesthetized with 0.4 mg/kg, s.c. medetomidine (Domitor vet 1 mg/mL, Orion Pharma, Espoo, Finland) and 20 mg/kg s.c. ketamine (Ketaminol vet 50 mg/mL, Pfizer Oy Animal Health, Espoo, Finland). Mydriasis was induced with topical tropicamide eyedrops (Oftan Tropicamid 5 mg/mL, Santen Pharmaceutical Co., Ltd., Tampere, Finland) and the ocular surface was anesthetized with oxybuprocaine eye drop (Oftan Obucain 4 mg/mL, Santen Pharmaceutical Co., Ltd., Tampere, Finland). Thereafter, intravitreal injection of triamcinolone acetonide (100 μL) was given with a 31G needle and syringe. After injection, the eyes were moisturized with carbomer gel (Viscotears 2 mg/g, Dr. Gerhard Mann chem.-pharm. Fabrik GmbH, Berlin, Germany) and the anesthesia was antagonized with 1 mg/kg atipamezole s.c. (Antisedan vet 5 mg/mL, Orion Pharma, Espoo, Finland).

After the second injection, IOP was allowed to settle for 10 days before the experiments were performed. The IOP remained on constant hypertensive level for about 35 days before it started to decrease toward normal levels. Induction of ocular hypertension is presented in Appendix Figure 3. The IOP-lowering effects of the molecules were investigated sequentially, each test initiating once baseline IOP levels was attained. Molecules with low melanin binding were investigated first (order: A22, dorzolamide, A12 and A01), thereby avoiding possible interference.

#### 2.5.3 IOP Measurements

The IOP levels in the rabbits were measured with a tonometer (Tonometer Pro, Icare Finland Ltd.). After baseline IOP measurements, a single eye drop (25 μL) was applied to the left eye, whereas the right eye was not treated. After eye drop instillation, the eyelids were kept open manually for 1 min. The IOP was measured from both eyes of the rabbits at 0.5, 1, 2, 3, 4, 5, 6, 7, and 8 h after eye drop instillation. In the case of the pigmented rabbits, the measurements were continued until 10 h postinstillation, and further until the IOP had returned to the normal baseline.

The CAIs were tested in the same rabbits to minimize interindividual data variation. Washout periods of at least 3 days were used between instillation of different compounds. The experiments started always at 8 a.m. to eliminate bias due to diurnal IOP fluctuation. At each time point, the pressure was measured three times with the same eye, and the average values were calculated and used in data analysis.

### 2.6 Pharmacokinetic Rat Study

#### 2.6.1 Radiolabeling of CAI **A01** and Preparation of Solution

To conduct the pharmacokinetic study, bromo-containing CAI **A15** (molecular structure presented in Table 1) was conveniently converted into [^3^H]-**A01** through catalytic dehalogenation in tritium gas. **A15** (3.05 mg, 8.2 μmol) was dissolved in dimethylformamide (0.3 mL) and exposed to 99 mbar of tritium gas in a tritium manifold system for 1.5 h in the presence of 10% palladium on carbon (0.57 mg). Following filtration, the reaction mixture underwent double lyophilization with 1 mL ethanol to eliminate labile tritium. LC–MS analysis revealed approximately 50% conversion according to UV-detection. The crude material was purified via high-performance liquid chromatography, employing a Reprosil-Pur C18-HD column (250 × 10 mm, 5 μm), with a mobile phase comprising 40% acetonitrile in 50 mM ammonium hydrogen carbonate. Purified fractions were subsequently combined, subjected to solid-phase extraction, and eluted with ethanol. Radiochemical analysis indicated a purity exceeding 97%, with a molar activity of 0.29 TBq/mmol (7.9 Ci/mmol) and a total activity of 850 MBq (23 mCi). The solution of 1% **A01** (10 mg/mL) was prepared similarly as in the IOP study (see Table 1) except that the solution contained also 72 μg/mL of [^3^H]–**A01** (specific activity 27.21 mCi/mg). The solution was prepared freshly for each experiment.

#### 2.6.2 In Vivo Pharmacokinetic Rat Study

Seven μL of **A01** solution (10 mg/mL including 72 μg/mL of ^3^H–**A01**) was topically applied to both eyes of albino and pigmented rats. Rats were euthanized with carbon dioxide and cervical dislocation. The eyes (*n* = 4) of albino rats were collected 0.5, 2, 4, and 7 h after treatment. The eyes of pigmented rats were dissected 0.5, 2, 4, 7, 24, 48, 72, 144, and 240 h after eye drop instillation. The eyes were enucleated and dissected immediately after euthanasia. Iris-ciliary body was dissected, snap frozen on dry ice, and stored at −80 °C until analysis. The analyses were conducted within 24 h from tissue dissection.

Bound and unbound radioactivity of tritiated **A01** was determined from dissected iris-ciliary body samples. First, the tissues were mechanically homogenized and diluted with 0.9% sodium chloride (weight ratio 35:1). Then, cellular unbound **A01** was released from the iris-ciliary body cells by freezing and thawing the solutions three times with dry ice and a water bath (37 °C). Thereafter, the supernatant and pellet were separated by centrifuging the samples for 10 min at 13,000 rpm. The pellets were dissolved by using 60 min incubation in 100 μL of Solvable (PerkinElmer, Waltham, MA, USA). The samples were then bleached with 10 μL of hydrogen peroxide for 30 min. The pellet volume was calculated as the difference between the total and supernatant volumes.

Radioactivity of the pellets and supernatants (counts per minute) was measured with a liquid scintillation counter (2450 MicroBeta^2^, PerkinElmer, Singapore Pte. Ltd.). After subtracting the background radioactivity, the measured net counts per minute (cpm) were converted to disintegrations per minute (dpm) taking into account color quenching and device efficiency. Standard curves were generated for each matrix (albino and pigmented supernatants and pellets; Appendix Figure 1). Finally, unbound, bound, and total **A01** concentrations (ng/mg) in the iris-ciliary body of the rabbits were calculated using radioactivity (nCi) per μg of instilled **A01** in the eye drops. Signal to noise ratios of measured pellet and supernatant samples in liquid scintillation counting are shown in Appendix Table 3.

## 3 Results

### 3.1 Melanin Binding of the CAIs In Vitro

To discover high melanin binding CAI-II compounds, we screened a set of previously developed compounds, representing several CAI classes: benzenesulfonamides **A01–A30**, 5-membered heterocyclic sulfonamides **B01–B11**, as well as mono- and disubstituted sulfamides **C01–C22** (Appendix Table 1). Among the new CAI molecules, one was classified as a high binder, 34 as intermediate binders, and 28 as low or nonbinders. In addition, 5 control molecules (penicillin G, brimonidine, dorzolamide, atropine, brinzolamide) were screened for in vitro melanin binding using MST. The melanin binding affinity of control molecules and three molecules selected for in vivo IOP-lowering studies are shown in Table 2. The *K*_d_ values of penicillin G and atropine were consistent with earlier results measured with microscale thermophoresis.^20^ Brimonidine was classified as a high melanin binder (*K*_d_ < 65 μM), dorzolamide as an intermediate binder (*K*_d_ 65–650 μM), and brinzolamide as a low binder (*K*_d_ > 650 μM).

**Table 2:**
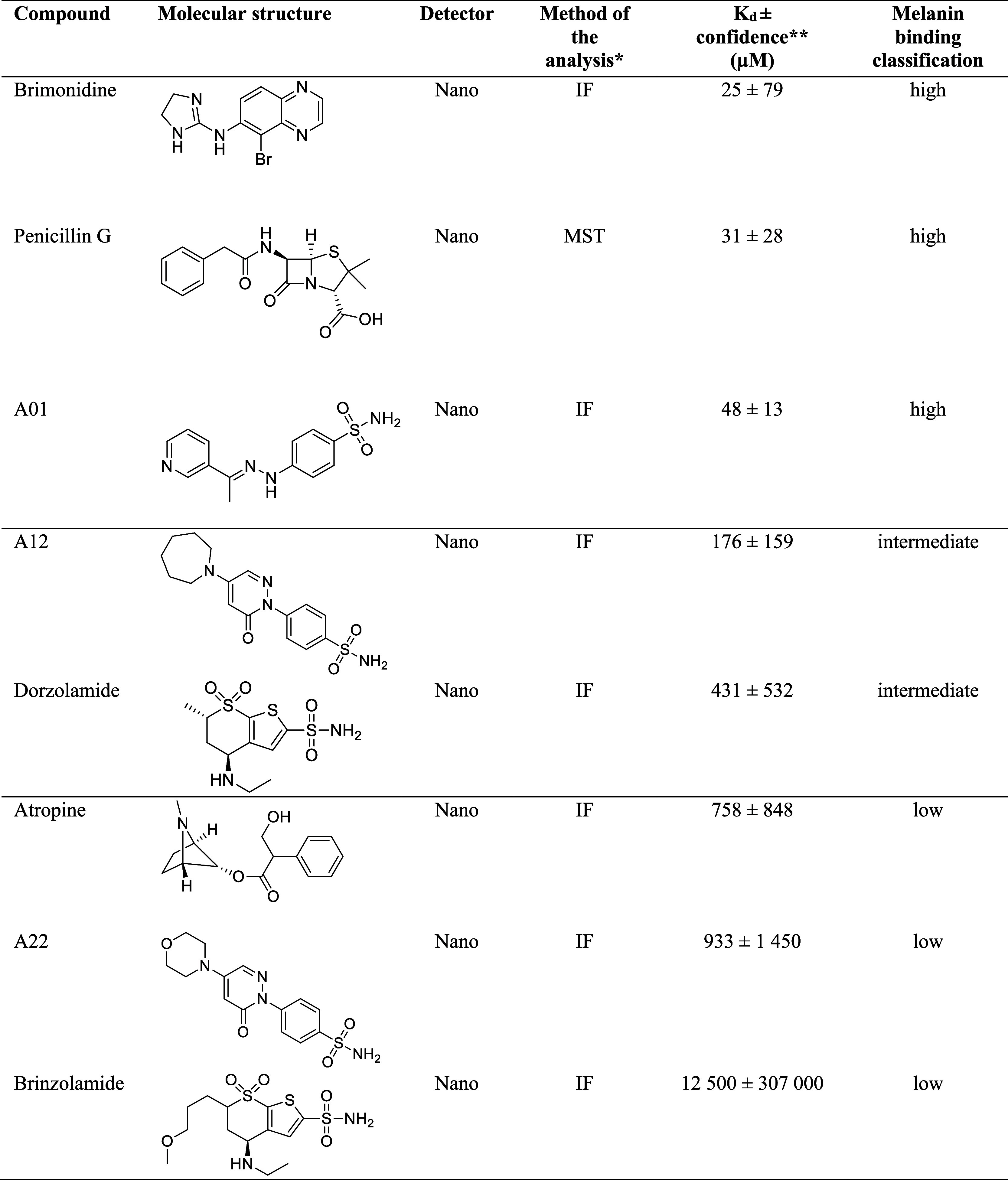
Melanin Binding Data and Molecular Structures of Novel CAI Compounds Selected for In Vivo Efficacy Studies and Control Molecules

a MST mode was utilized when *K*_d_ value could not be determined with IF mode.

b The 68% confidence values for *K*_d_ were obtained within the MO. Affinity Analysis Software.

### 3.2 Affinity to Human Carbonic Anhydrase II In Vitro

Affinity of selected CAIs on human carbonic anhydrase II is shown in Table 3. The experimental CAIs showed affinities in the range 10^–8^ to 10^–7^ M, whereas control compounds had affinities of 10^–9^ to 10^–8^ M. Table 3 presents also rate constants for association and dissociation as obtained from the surface plasmon resonance. Potency of all compounds (either *K*_i_ values from literature or *K*_d_ values from surface plasmon resonance) are presented in Appendix Table 1). CAI potency of most compounds (in the nM range) was considered to be adequate^12−19^ (Appendix Table 1).

**Table 3:** Parameters for the Interaction of Different CAIs with Human Carbonic Anhydrase II

CAI	*k*_a_ (10^6^ M^–1^ s^–1^)	*k*_d_ (10^–2^ s^–1^)	*K*_D_ (nM)
A01	0.57 ± 0.33	1.45 ± 0.15	36.90 ± 23.39
A05	0.61 ± 0.25	1.70 ± 0.25	31.50 ± 9.19
A07	0.24 ± 0.05	1.78 ± 0.40	75.57 ± 11.22
A15	0.43 ± 0.25	1.25 ± 0.43	46.00 ± 29.81
A17	0.13 ± 0.05	1.27 ± 0.11	113.30 ± 50.67
A19	0.15 ± 0.04	2.25 ± 0.02	161.33 ± 40.96
acetazolamide	1.04 ± 0.46 × 10^6^	1.24 ± 0.47 × 10^–2^	13.15 ± 3.44
dorzolamide	1.18 ± 0.62 × 10^6^	0.56 ± 0.12 × 10^–2^	6.64 ± 3.86

### 3.3 Experimental CAIs Have Adequate Predicted Corneal and Conjunctival Permeability

Topically applied CAIs must absorb across the cornea or conjunctiva to the ciliary body to exert an IOP response. Apparent permeability coefficients of the CAIs were estimated computationally for the porcine cornea and conjunctiva. Since calculated permeability coefficients of the CAIs were comparable with dorzolamide and brinzolamide in cornea (1.75–3.16 × 10^–7^ cm/s) and conjunctiva (1.77–2.80 × 10^–6^ cm/s) (Appendix, Table 2, calculated with Appendix eqs 1 and 2), we do not expect that membrane permeability would significantly limit their efficacy in vivo. Since CAI potency and permeation of the compounds seem adequate, selection of the CAIs for in vivo studies was done based on the melanin binding data. A high melanin binding CAI **A01** was tested head-to-head with other benzenesulfonamide-based CAI-II derivatives **A12** and **A22** with intermediate and low affinity to melanin, respectively.

### 3.4 Melanin Binding CAIs Show Extended IOP-Lowering Effects

**Figure 1:**
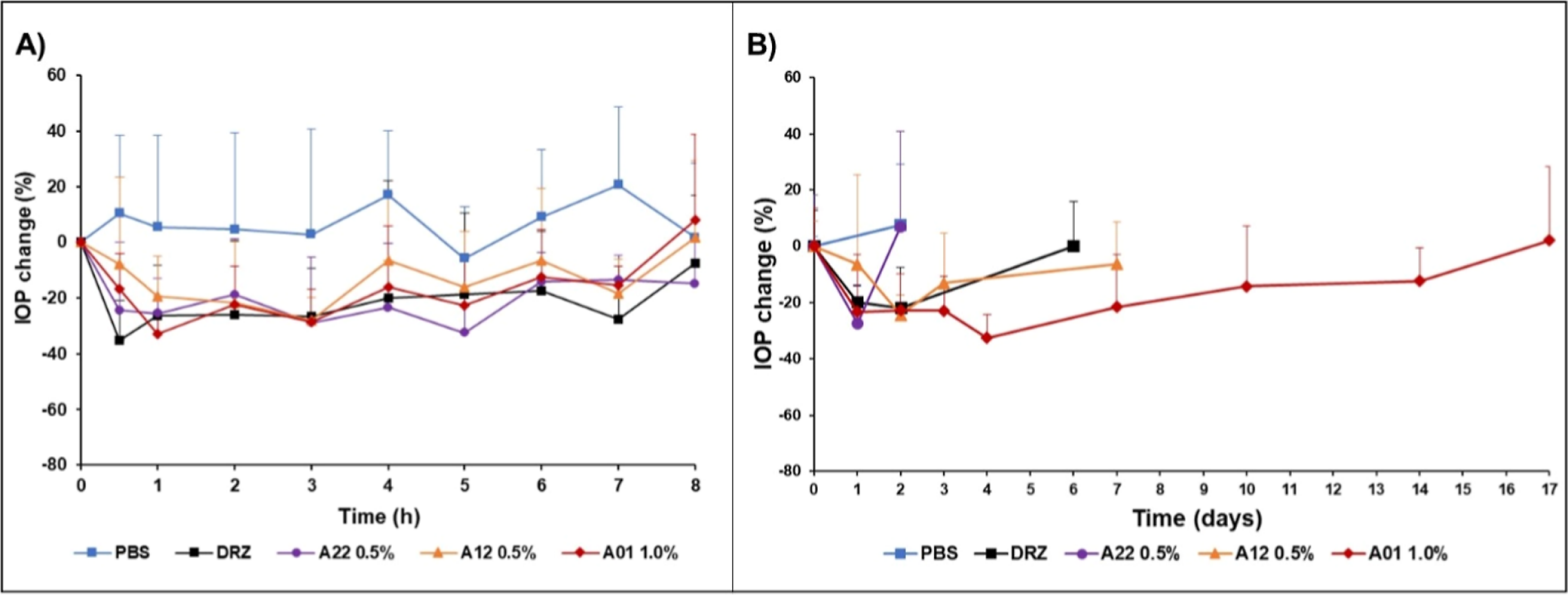
IOP-lowering effect of the CAIs in albino (A) and pigmented (B) rabbits (*n* = 5 animals/group). Relative mean (±S.D.) changes of pressure compared to pretreatment levels are shown. PBS = phosphate buffered saline, DRZ = dorzolamide, **A22** = low melanin binding CAI, **A12** = intermediate melanin binding CAI, and **A01** = high melanin binding CAI. (A) In albino eyes, AUC_0-last_ values of IOP responses were statistically tested with Friedman Repeated Measurement Analysis of Variance on Ranks. The statistical test against placebo (paired *t*-test with each molecule separately) shows statistically significant difference in AUC_0-last_ values of A01 (*P* = 0.005, *t* = −5.569 with 4 degrees of freedom), and dorzolamide (*P* = 0.043, *t* = −2.923 with 4 degrees of freedom), but not for A12 (*P* = 0.052, *t* = −2.742 with 4 degrees of freedom) and A22 (*P* = 0.074 *t* = −2.406 with 4 degrees of freedom). (B) In pigmented eyes, the AUC_0-last_ of IOP response of **A01** was significantly greater (*P* < 0.001, *t* = 7.509) than that of dorzolamide (one-way repeated measures analysis of variance with Bonferroni *t*-test; *F* = 30.755, power of the performed test with alpha 0.050:1.000). Significant difference was not seen between dorzolamide and **A12** (*P* = 1.0, *t* = 0.388) or between dorzolamide and **A22** (*P* = 1.0, *t* = 0.571).

The CAIs were tested first as 0.1% eye drops in normotensive albino rabbits (Appendix Figure 2), but the IOP-lowering effects were weaker than those of 2% dorzolamide (Trusopt). Thus, CAI concentrations of 0.5 and 1.0% were used in the eye drops. All tested CAIs, including dorzolamide, decrease IOP for 4–8 h in albino rabbits (*E*_max_ = 28–37% decrease from the baseline at 0.5–3 h postinstillation) (Figure 1A). In the albino animals, the duration of the IOP response was not affected by melanin-binding properties of the compounds (Figure 1A). Likewise, areas under the response vs time curves of the new CAIs (Table 4) did not differ from that of dorzolamide in albino rabbits (Friedman Repeated Measurement Analysis of Variance on Ranks; 3 degrees of freedom, *P* = 0.050). The values for the areas under the IOP response versus time curve (AUC) represent drug exposure to the CA-II in the rabbit ciliary body.

**Table 4:** IOP Decreasing Effects of CAIs in Albino and Pigmented Rabbits^a^

compound	AUC_0-last_ ± SD in albino rabbits (mmHg x h)	AUC_0-last_ ± SD in pigmented rabbits (mmHg × h)	ratio (AUC_pigm_/AUC_albino_)
dorzolamide	15.55 ± 10.64	202.35^1^ ± 122.60	13.0
A01	19.95 ± 7.16	1704.90^2^ ± 530.82	85.5
A12	13.80 ± 10.06	124.65^3^ ± 79.15	9.0
A22	10.50 ± 8.73	88.10 ± 63.04	8.4

a Areas under the IOP response versus time curve are presented. AUC_0-last_ = area under the curve for the decrease of IOP (mmHg) vs time (hours). The area was determined relative to the initial baseline at the time of eyedrop instillation until the last IOP measurement time*.* The AUC_0-last_ presenting IOP-lowering effect (decrease in mmHg vs time as compared to the baseline) of each molecule in pigmented and albino eyes was compared for each molecule with Welch’s (unequal variances) *t*-test using the SigmaPlot 15.0 program: ^1^statistically significant *P* = 0.03 (*t* = −3.036 with 4.06 degrees of freedom), ^2^statistically significant *P* = 0.003 (*t* = −6.348 with 4.001 degrees of freedom), ^3^statistically significant *P* = 0.04 (*t* = −2.778 with 4.129 degrees of freedom).

Since the baseline IOP of pigmented rabbits was only 9 mmHg, ocular hypertension was induced with intravitreal triamcinolone acetonide injections (Appendix Figure 3). In these hypertensive pigmented rabbits, the IOP-lowering effects of the CAIs lasted for more than 24 h (Figure 1B). The high melanin binder compound (**A01**) reduced the IOP for 14 days after instillation of a single eye drop (Figure 1B). The IOP-lowering effect of topical CAIs during the first 10 h after instillation is presented in Appendix Figure 4. Duration of IOP-lowering effects of the CAIs in pigmented rabbits was prolonged with increasing melanin binding (**A22** < **A12** < **A01**) (Figure 1B).

In pigmented rabbits, the response AUC values of CAIs were remarkably elevated with increasing melanin binding (Table 4). Melanin binding compounds (**A01, A12,** and dorzolamide) showed significantly higher AUC response values in the pigmented rabbits than in the albino rabbits. In the case of **A01** (high melanin binder), response AUC was 85 times higher than in the pigmented rabbits compared with albino animals. The response AUC values were elevated with increasing melanin binding of the CAI compound (**A22 < A12** < **A01**).

### 3.5 Extended CAI Retention in the Iris-Ciliary Based on Melanin Binding

**Figure 2:**
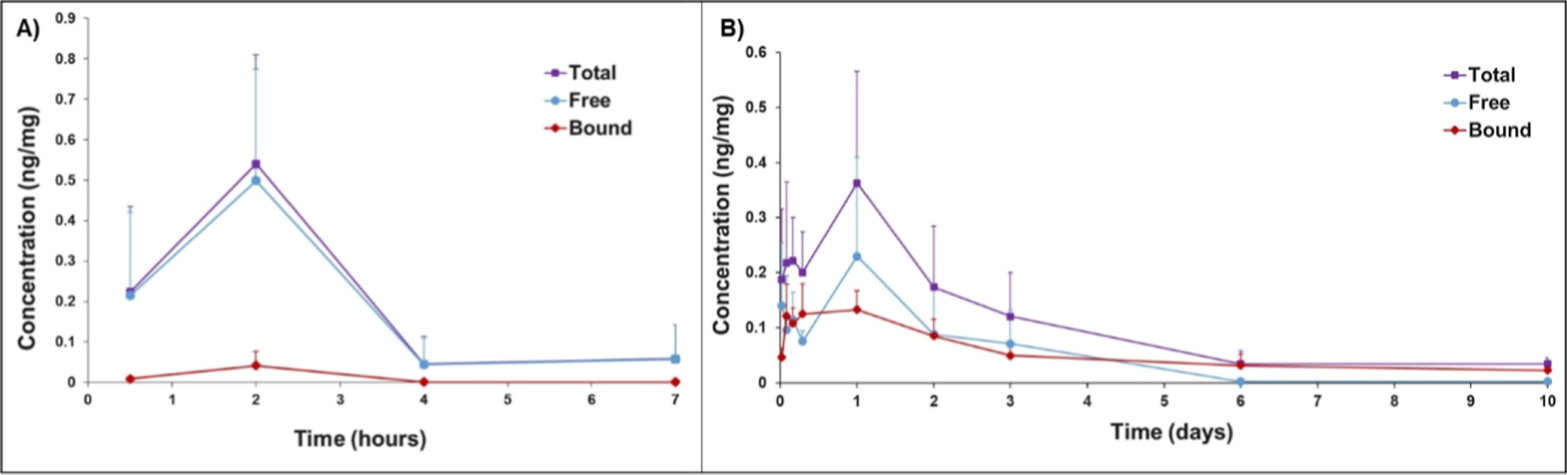
Total **A01** concentrations (ng/mg) (purple) in albino (A) and pigmented (B) iris-ciliary body tissues after instillation of a single topical eye drop (*V* = 7 μL; **A01** concentration = 10 mg/mL) to rats. Unbound (blue) and bound **A01** concentrations (red) are also shown. The data show mean ± SD (*n* = 4) values. There is no statistically significant difference in *C*_max_ values of total drug in albino (*t*_max(total drug)_ = 2 h) and pigmented (*t*_max(total drug)_ = 24 h) eyes (two-tailed *P*-value = 0.398, *t* = 0.910 with 6 degrees of freedom, tested with Student’s *t*-test), whereas the difference in bound drug concentrations (*C*_max(bound drug)_) between albino (*t*_max_ = 2 h) and pigmented (*t*_max_ = 24 h) eyes was significant (two-tailed *P*-value = 0.0172, *t* = −3.264 with 6 degrees of freedom, tested with Student’s *t*-test).

Ocular pharmacokinetic study of the high melanin binder (**A01**) was conducted in albino and pigmented rats using instillation of a single eyedrop (10 mg/mL), spiked with ^3^H–**A01** (72 μg/mL). The total **A01** concentration (bound + unbound) in albino iris-ciliary body tissues peaked at 2 h postdosing (*C*_max_ = 0.54 ng/mg) and declined rapidly to ≈0.05 ng/mg (Figure 2A). The levels of **A01** were maintained longer in the pigmented iris-ciliary body: *C*_max_ of 0.36 ng/mg was reached at 24 h and slow elimination continued at least for 10 days (Figure 2B).

Since the CAI response depends on the concentrations of unbound drug, we analyzed unbound and bound concentrations of **A01** in the iris-ciliary body of albino and pigmented rats. In the albino iris-ciliary body, the free **A01** concentration was relatively high at 2 h postdosing (*C*_max,unbound_ = 0.50 ng/mg), but declined rapidly by 4 h (to ≈0.05 ng/mg) (Figure 2A). The bound **A01** concentrations in the iris-ciliary body were low (<0.05 ng/mg).

**Figure 3:**
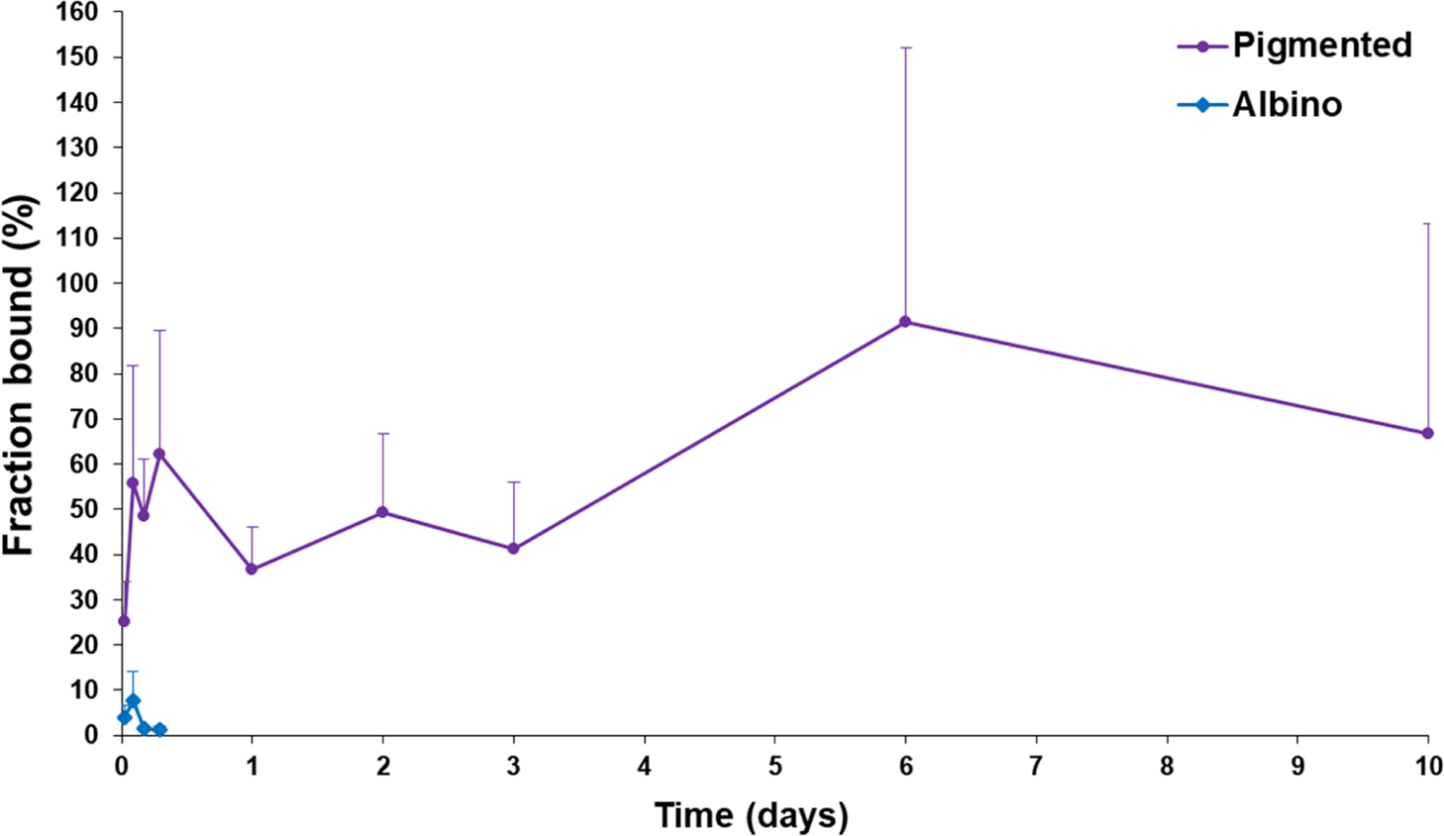
Bound fraction of high melanin binder CAI (**A01**) in albino and pigmented rat iris-ciliary body after single eye drop administration. Means ± SD (*n* = 4) are presented.

In the pigmented iris-ciliary body, the bound **A01** concentration reached ≈0.5 ng/mg at 2 h after administration and then decreased slowly during 10 days (*C*_10 days_ = 0.02 ng/mg) (Figure 2B). Interestingly, the unbound levels of **A01** were extended in the pigmented iris-ciliary body for at least 3 days, decreasing below the quantitation limit at 6 days postinstillation. The bound fraction of **A01** remains rather constant (37–62%) for 72 h in pigmented tissue and rises later to about 90%, whereas it remained below 10% in albino iris-ciliary body (Figure 3).

The AUC values for **A01** concentration vs time curves are presented in Table 5. The total **A01** exposure (bound + unbound drug) was 18 times higher in the pigmented iris-ciliary body than in the albino counterpart. Even 150-fold higher bound drug levels were seen in the pigmented iris-ciliary body as compared to the bound levels in the albino tissue. Furthermore, the AUC value of the unbound drug was 9.3 times higher in the pigmented than albino iris-ciliary body.

**Table 5:** Exposure of CAI **A01** in Albino and Pigmented Iris-Ciliary Body of Rats (AUC) after Instillation of a Single Topical Eye Drop^a^

	AUC_0-last_ ((ng/mg) × h) in albino tissues	AUC_0-last_ ((ng/mg) × h) in pigmented tissue	exposure ratio (AUC_pigm_/AUC_albino_)
total **A01**	1.37	24.72	18.0
bound **A01**	0.09	12.73	149.8
unbound **A01**	1.29	11.98	9.3

a Bound and unbound concentrations were separately quantitated and used for AUC calculations.

## 4 Discussion

Melanin binding of some clinical drugs has been known for decades,^3,4,27,28^ but only recently systematic studies have been performed to understand the chemical drivers of binding and to reveal the rationale of the pharmacological impact of melanin binding.^2,20,29−32^ Chemical features of melanin binding^32^ and correlation between in vitro and in vivo melanin binding^2^ have set the stage for the use of melanin binding as a tool for targeted drug delivery. In this study, we demonstrate for the first time how melanin binding can be used as a powerful drug design tool in the discovery of small molecular ocular drugs with targeted tissue disposition. We show that high melanin binding of CAI-II compound results in 2 weeks’ duration of IOP-lowering effect in the eyes of pigmented rabbits after administration of a single eyedrop.

**Figure 4:**
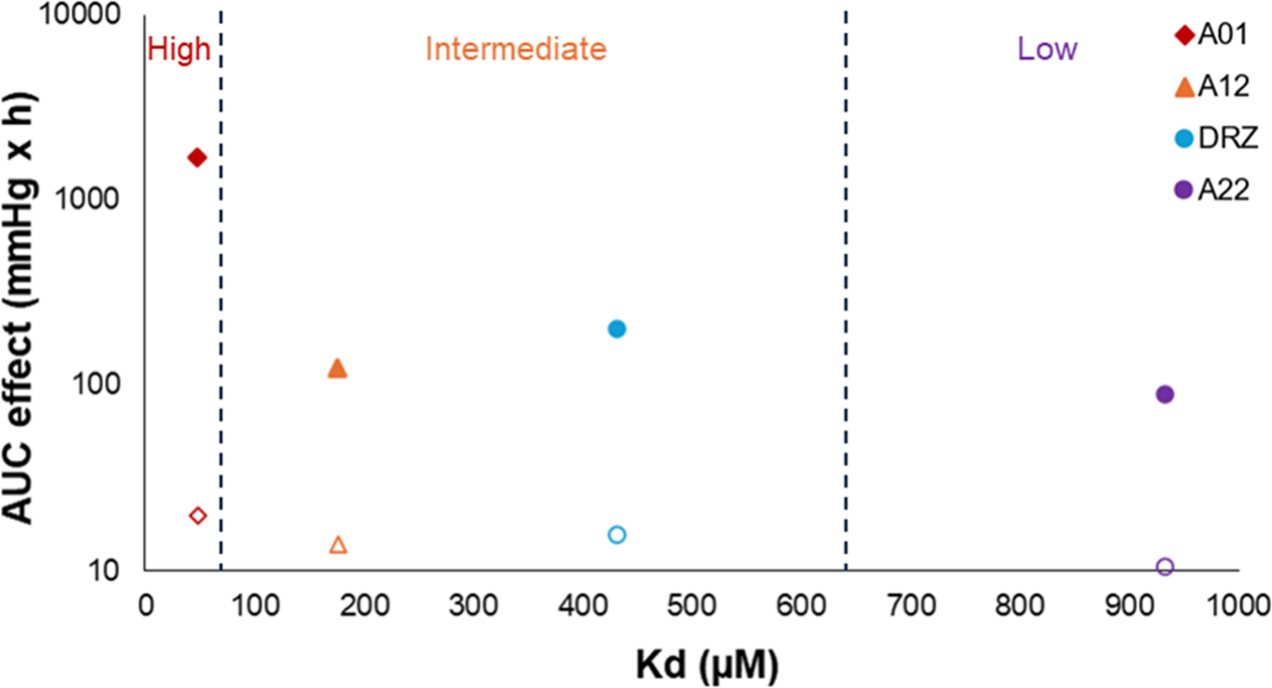
AUC of the IOP-lowering effect in pigmented rabbits presented in logarithmic scale and melanin binding affinity (*K*_d_) of the CA-II inhibitors with different melanin-binding properties (**A01** = CAI with high melanin binding, **A12** = CAI with intermediate melanin binding, DRZ = dorzolamide, and **A22** = CAI with low melanin binding). The filled symbols show the response AUC results in pigmented rabbits and the open symbols represent the results in albino rabbits.

In our study, 63 CAIs^12−19^ (Appendix Table 1) were studied for their melanin binding with microscale thermophoresis. Dorzolamide, high (**A01**), intermediate (**A12**), and low (**A22**) melanin binder compounds were selected for the IOP-studies in rabbits. Pharmacokinetic and pharmacodynamic differences were dramatic between albino and pigmented rabbits, demonstrating the pharmacological importance of melanin binding in the ciliary body. In the albino eyes, the IOP-lowering effects of the new molecules (0.5–1% solutions) and dorzolamide (2%) were short-lived (<8 h). Much longer responses were seen in the pigmented rabbits, and the duration of effects depended strongly on melanin binding of the CAI (Figures 1 and 4). Most remarkably, the high melanin binder (**A01**) resulted in 2 weeks’ decrease of IOP after a single eyedrop instillation.

Even the low melanin binding CAI (**A22)** showed some prolongation in the IOP-lowering effect in the pigmented eyes. **A22** has comparable melanin binding affinity with atropine^20^ and pilocarpine,^33^ which show longer mydriatic (atropine) and miotic (pilocarpine) effects in pigmented rabbits than in the albino rabbits.^3,4^ Melanin concentrations in the ocular tissues (∼30 μg/mg in the human ciliary body and ∼100 μg/mg in human iris^33^) are significantly higher than the melanin levels that are used in in vitro assays (0.5–5 μg/mg).^20^ Therefore, in vivo impact of melanin binding may be more pronounced than in vitro, explaining the prolonged IOP effect of **A22**, atropine,^3^ and pilocarpine.^3,4^

In IOP-lowering studies, the albino animals were normotensive and pigmented animals were hypertensive. The model difference should not affect the results because the baseline IOP levels in triamcinolone-treated pigmented rabbits and normotensive albino rabbits were similar (on average, ∼11 mmHg). Regardless of melanin binding, we did not see any differences in IOP responses in albino rabbits, while the differences are obvious in the pigmented eyes. Additionally, pharmacokinetic analyses in albino and pigmented rats support prolonged retention in the pigmented iris-ciliary body as the reason for prolonged activity. Therefore, it is unlikely that the improved efficacy of high melanin binder CAIs would be due to some other reasons in triamcinolone treated animals.

The total iris-ciliary body exposure to **A01** after a single eye drop administration was 18 times higher in the pigmented than in the albino rat eye. Furthermore, the AUC of free **A01** was 9.3 times higher, and the bound AUC was 150 times higher in the pigmented than in albino iris-ciliary body. Our data demonstrate that the melanin bound depot can release drug over long times, maintaining also the unbound drug at pharmacologically relevant levels, thereby explaining the observed prolonged pharmacological effects. The possible species differences in the pharmacokinetics of topically applied small molecule drugs in rats and rabbits have not been discussed in the literature. However, the duration of IOP-responses of dorzolamide were reported modestly longer in albino rabbits^36,37^ (∼6 h) compared to albino rats^38^ (∼4 h), which may indicate the faster drug elimination in rat than rabbit eyes.

This is the first published study that shows analyses of bound and unbound drug fractions in vivo in pigmented and albino tissues. Understanding the equilibrium between bound and unbound drugs is essential for understanding the impact of melanin binding on the extent and duration of drug response. Earlier, Shinno et al.^35^ estimated the concentrations of bound and unbound brimonidine in the retina-choroid by using static in vitro equilibrium studies for estimating unbound brimonidine levels in vivo. However, the binding equilibrium in a static in vitro experiment is different from a dynamic in vivo situation with active clearance factors.

In accordance with our results, Jakubiak et al. (2019)^2^ showed that the high or extreme melanin binder compounds (e.g., levofloxacin and sunitinib) are retained for weeks in the pigmented eyes of rat after intravenous injections, whereas the intermediate binders may retain for days (e.g., timolol). It should be noted that in addition to the melanin binding, also drug potency and membrane permeability may have an influence on the extension of drug response.^1^ With potent compounds, effective concentrations of unbound drug can be maintained even when most of the drug is bound to melanin, whereas melanin binding may render the unbound concentrations of low potency compounds to too low levels.^1^ Threshold concentrations for activity [e.g., receptor binding affinity (*K*_d_), enzyme inhibition constants (*K*_i_) or minimum inhibitory concentrations (MIC)] vary over a wide range. For example, affinities for receptor binding or enzyme inhibition are often in the range of 10^–9^ to 10^–8^ M (e.g., timolol, atropine, brinzolamide), whereas MIC values of antibiotics are usually at the levels of 10^–6^ to 10^–4^ M (e.g., ampicillin, gentamycin, chloramphenicol). Extended drug response has been associated with melanin binding of potent drugs. For example, the mydriatic effect of topical atropine eye drops in pigmented rabbits (>96 h) lasted two times longer than in albino rabbits (43.5 h).^3,40^ On the other hand, Nagata et al. (1993) observed stronger IOP-lowering effects with timolol, adrenaline, and pilocarpine in albino rabbits than in pigmented rabbits.^39^ Alpha-2-agonist brimonidine is a melanin binding compound that accumulates in the pigmented tissues,^47^ but the duration of the IOP-lowering effect in albino and pigmented eyes and in normotensive or hypertensive rabbits has been reported to be at the same range.^41−46^ However, extension to the IOP response of intracameral brimonidine was achieved by conjugating the drug to a melanin binding peptide.^31^ Overall, melanin binding can lead to prolonged drug response, as shown also in this study for topical CAIs. Since the drug response is affected by many factors, high melanin binding of a compound does not necessarily lead to its prolonged pharmacological effect.

Inhibition constants of many CAI-II compounds are in the nanomolar and subnanomolar range (Appendix Table 1). The unbound concentrations of **A01** in the pigmented rat iris-ciliary body were ≈10–800 nM, adequate levels for exerting inhibition of carbonic anhydrase II (*K*_d_ = 36.9 nM). Both high potency and melanin binding are needed to achieve targeted delivery and prolonged efficacy in pigmented tissue. The free drug concentrations are also affected by the membrane permeability of the drug (i.e., melanosome and plasma membranes), since these factors also influence the overall drug elimination from melanin-containing tissue.^48^

Our study demonstrates for the first time how melanin binding can be used to design a small molecular drug that is capable of ciliary body homing and produces prolonged IOP response of nearly 2 weeks after instillation of a single eye drop. The only previous studies that use melanin binding as drug design criterion were published in 2023.^31,49^ In those studies, peptides were screened for melanin binding and the optimized peptide were conjugated to sunitinib^49^ or brimonidine.^31^ The sunitinib conjugate was studied after topical administration in rats, resulting in improved intraocular drug residence time and neuroprotective activity in a rat model of optic nerve injury.^49^ In the other study, the conjugate and brimonidine were tested in vivo as intracameral injections that resulted in IOP decrease of 18 days (the conjugate) and 7 days (brimonidine) in the eyes of pigmented rabbits.^31^ Although positive results were obtained, it should be remembered that intracameral injections can be conducted only in the clinics and, therefore, duration of action for such injections should be preferably 3–6 months.^50,51^ Prolonging topical administration frequency of CAI from 2 to 3 times a day to once a week or once in 2 weeks would be a major improvement in clinical treatment. Reduced dosing frequency would also decrease overall drug load in the chronic drug treatment, possibly improving the safety and adherence of the treatment.^52^ Currently, low adherence (<50% of glaucoma patients) is one of the biggest challenges in glaucoma treatment.^53−55^

The prolonged IOP-responses of **A01**, shown in pigmented rabbits, are likely in the human eyes despite some species differences. Permeabilities of 2 methazolamide and ethoxzolamide were similar in rabbit and human cornea, whereas benzolamide and bromacetazolamide had higher permeation in the human cornea.^56^ In addition to corneal permeability, blinking rate and conjunctival permeation may affect ocular bioavailability.^56^ The prolongation of the IOP response of **A01** in pigmented rabbits is not directly translatable to human eyes, but significant prolongation in human eyes is likely. Melanin concentrations show some species differences,^49,57^ but the melanin concentrations in the ciliary body of rabbit, rat, and human have not been compared. Menon et al.^34^ determined high melanin concentrations in human ciliary body (∼35 μg/mg) and iris (∼100 μg/mg). Thus, the prolonged effects of potent and high melanin binding CAI-II inhibitors are likely in the human eyes. The difference in the total melanin amount between blue and brown eyes has been shown to be insignificant.^34^ Thus, melanin drug depots can be used in patients with different eye colors except those with albinism. However, if there are response differences based on eye color, the dosing regimens can be adjusted easily by using eye color as a biomarker.

## 5 Conclusions

We demonstrate that melanin binding is a powerful design criterion for ocular drug development. Design of melanin binding drugs facilitates the development of tissue-targeted drugs with long retention and extended duration of action. We show that these principles can be utilized in the discovery of topically applied long-acting drugs for the treatment of glaucoma. The approach might be expanded also to the delivery of drugs to the posterior eye segment to treat pathologies of retina and choroid as ocular injections and systemic and topical delivery.

## Supporting Information Available

Molecular structures and descriptors of new CAI molecules; calculated permeability values of dorzolamide, brinzolamide, and experimental compounds in porcine cornea and conjunctiva; standard curves for quantitation of ^3^H–A01 in tissue pellets and supernatants; signal-to-noise ratios of the samples in liquid scintillation counting; equations for estimation of corneal and conjunctival permeability in porcine tissues; IOP responses of albino rabbits after 0.1% CAI eye drops and 2% dorzolamide; IOP profiles after two intravitreal triamcinolone injections into pigmented rabbits; IOP responses of albino rabbits during the first 10 h after instillation of experimental CAI eye drops and dorzolamide; and binding of CAIs to hCA-II as measured with a multiparametric surface plasmon resonance device (PDF)

## Abbreviations

AUC: area under the curve
AUC_0-last_: area under the curve from time 0 to the last sampling point
CA: carbonic anhydrase
CAI: carbonic anhydrase inhibitor
DRZ: dorzolamide
*E*_max_: maximum effect
IF: initial fluorescence
IOP: intraocular pressure
*K*_i(hCA-II)_: inhibition constant against human CA-II
*K*_d_: dissociation constant
MST: microscale thermophoresis
PBS: phosphate buffered saline
s.c.: subcutaneous
TA: triamcinolone acetonide

## References

[ref1] Rimpelä A. K.; Reinisalo M.; Hellinen L.; Grazhdankin E.; Kidron H.; Urtti A.; del Amo E. M. Implications of melanin binding in ocular drug delivery. Adv. Drug Delivery Rev. 2018, 126, 23–43. 10.1016/j.addr.2017.12.008. 29247767

[ref2] Jakubiak P.; Cantrill C.; Urtti A.; Alvarez-Sánchez R. Establishment of an In Vitro-In Vivo Correlation for Melanin Binding and the Extension of the Ocular Half-Life of Small-Molecule Drugs. Mol. Pharmaceutics 2019, 16, 4890–4901. 10.1021/acs.molpharmaceut.9b00769. 31670965

[ref3] Salazar M.; Shimada K.; Patil N. P. Iris pigmentation and atropine mydriasis. J. Pharmacol. Exp. Ther. 1976, 197, 79–88. 1263134

[ref4] Urtti A.; Salminen L.; Kujari H.; Jäntti V. Effect of ocular pigmentation on pilocarpine pharmacology in the rabbit eye. II. Drug response. Int. J. Pharm. 1984, 19, 53–61. 10.1016/0378-5173(84)90132-7.

[ref5] Menon I. A.; Trope G. E.; Basu P. K.; Wakeham D. C.; Persad S. D. Binding of timolol to iris-ciliary body and melanin: An in vitro model for assessing the kinetics and efficacy of long-acting antiglaucoma drugs. J. Ocul. Pharmacol. 1989, 5, 313–324. 10.1089/jop.1989.5.313. 2576434

[ref6] Pitkänen L.; Ranta V.-P.; Moilanen H.; Urtti A. Binding of betaxolol, metoprolol and oligonucleotides to synthetic and bovine ocular melanin, and prediction of drug binding to melanin in human choroid-retinal pigment epithelium. Pharm. Res. 2007, 24, 2063–2070. 10.1007/s11095-007-9342-0. 17546409

[ref7] Mishra C. B.; Tiwari M.; Supuran C. T. Progress in the development of human carbonic anhydrase inhibitors and their pharmacological applications: Where are we today?. Med. Res. Rev. 2020, 40, 2485–2565. 10.1002/med.21713. 32691504

[ref8] Hoyng P. F. J.; van Beek L. M. Pharmacological Therapy for Glaucoma: A Review. Drugs 2000, 59, 411–434. 10.2165/00003495-200059030-00003. 10776828

[ref9] Naageshwaran V.; Ranta V.-P.; Gum G.; Bhoopathy S.; Urtti A.; del Amo E. M. Comprehensive Ocular and Systemic Pharmacokinetics of Brinzolamide in Rabbits After Intracameral, Topical, and Intravenous Administration. J. Pharm. Sci. 2021, 110, 529–535. 10.1016/j.xphs.2020.09.051. 33035542

[ref10] McClelland J. F.; Bodle L.; Little J.-A. Investigation of medication adherence and reasons for poor adherence in patients on long-term glaucoma treatment regimes. Patient Prefer. Adherence 2019, 13, 431–439. 10.2147/PPA.S176412. 31496662 PMC6697779

[ref11] Reilly J.; Williams S. L.; Forster C. J.; Kansara V.; End P.; Serrano-Wu M. H. High-Throughput Melanin-Binding Affinity and In Silico Methods to Aid in the Prediction of Drug Exposure in Ocular Tissue. J. Pharm. Sci. 2015, 104, 3997–4001. 10.1002/jps.24680. 26524700

[ref12] Sharonova T.; Zhmurov P.; Kalinin S.; Nocentini A.; Angeli A.; Ferraroni M.; Korsakov M.; Supuran C. T.; Krasavin M. Diversely substituted sulfamides for fragment-based drug discovery of carbonic anhydrase inhibitors: synthesis and inhibitory profile. J. Enzyme Inhib. Med. Chem. 2022, 37, 857–865. 10.1080/14756366.2022.2051023. 35296197 PMC8933014

[ref13] Krasavin M.; Shetnev A.; Baykov S.; Kalinin S.; Nocentini A.; Sharoyko V.; Poli G.; Tuccinardi T.; Korsakov M.; Tennikova T. B.; Supuran C. T. Pyridazinone-substituted benzenesulfonamides display potent inhibition of membrane-bound human carbonic anhydrase IX and promising antiproliferative activity against cancer cell lines. Eur. J. Med. Chem. 2019, 168, 301–314. 10.1016/j.ejmech.2019.02.044. 30826507

[ref14] Krasavin M.; Shetnev A.; Sharonova T.; Baykov S.; Kalinin S.; Nocentini A.; Sharoyko V.; Poli G.; Tuccinardi T.; Presnukhina S.; Tennikova T. B.; Supuran C. T. Continued exploration of 1,2,4-oxadiazole periphery for carbonic anhydrase-targeting primary arene sulfonamides: Discovery of subnanomolar inhibitors of membrane-bound hCA IX isoform that selectively kill cancer cells in hypoxic environment. Eur. J. Med. Chem. 2019, 164, 92–105. 10.1016/j.ejmech.2018.12.049. 30594030

[ref15] Sapegin A.; Kalinin S.; Angeli A.; Supuran C. T.; Krasavin M. Unprotected primary sulfonamide group facilitates ring-forming cascade en route to polycyclic [1,4]oxazepine-based carbonic anhydrase inhibitors. Bioorg. Chem. 2018, 76, 140–146. 10.1016/j.bioorg.2017.11.014. 29175585

[ref16] Krasavin M.; Shetnev A.; Sharonova T.; Baykov S.; Tuccinardi T.; Kalinin S.; Angeli A.; Supuran C. T. Heterocyclic periphery in the design of carbonic anhydrase inhibitors: 1,2,4-Oxadiazol-5-yl benzenesulfonamides as potent and selective inhibitors of cytosolic hCA II and membrane-bound hCA IX isoforms. Bioorg. Chem. 2018, 76, 88–97. 10.1016/j.bioorg.2017.10.005. 29153590

[ref17] Krasavin M.; Korsakov M.; Ronzhina O.; Tuccinardi T.; Kalinin S.; Tanç M.; Supuran C. T. Primary mono- and bis-sulfonamides obtained via regiospecific sulfochlorination of N-arylpyrazoles: inhibition profile against a panel of human carbonic anhydrases. J. Enzyme Inhib. Med. Chem. 2017, 32, 920–934. 10.1080/14756366.2017.1344236. 28718328 PMC6445164

[ref18] Krasavin M.; Korsakov M.; Zvonaryova Z.; Semyonychev E.; Tuccinardi T.; Kalinin S.; Tanç M.; Supuran C. T. Human carbonic anhydrase inhibitory profile of mono- and bis-sulfonamides synthesized via a direct sulfochlorination of 3- and 4-(hetero)arylisoxazol-5-amine scaffolds. Bioorg. Med. Chem. 2017, 25, 1914–1925. 10.1016/j.bmc.2017.02.018. 28237553

[ref19] Kalinin S.; Kovalenko A.; Valtari A.; Nocentini A.; Gureev M.; Urtti A.; Korsakov M.; Supuran C. T.; Krasavin M. 5-(Sulfamoyl)thien-2-yl 1,3-oxazole inhibitors of carbonic anhydrase II with hydrophilic periphery. J. Enzyme Inhib. Med. Chem. 2022, 37, 1005–1011. 10.1080/14756366.2022.2056733. 35350949 PMC8973362

[ref20] Hellinen L.; Bahrpeyma S.; Rimpelä A.-K.; Hagström M.; Reinisalo M.; Urtti A. Microscale Thermophoresis as a Screening Tool to Predict Melanin Binding of Drugs. Pharmaceutics 2020, 12, 554 10.3390/pharmaceutics12060554. 32560065 PMC7355663

[ref21] Fan Q.; Cheng K.; Hu X.; Ma X.; Zhang R.; Yang M.; Lu X.; Xing L.; Huang W.; Gambhir S. S.; Cheng Z. Transferring Biomarker into Molecular Probe: Melanin Nanoparticle as a Naturally Active Platform for Multimodality Imaging. J. Am. Chem. Soc. 2014, 136, 15185–15194. 10.1021/ja505412p. 25292385 PMC4227813

[ref22] Rogez-Florent T.; Goossens L.; Drucbert A. S.; Duban-Deweer S.; Six P.; Depreux P.; Danzé P. M.; Goossens J. F.; Foulon C. Amine coupling versus biotin capture for the assessment of sulfonamide as ligands of hCA isoforms. Anal. Biochem. 2016, 51, 42–51. 10.1016/j.jpba.2017.01.023. 27485269

[ref23] Ramsay E.; Ruponen M.; Picardat T.; Tengvall U.; Tuomainen M.; Auriola S.; Toropainen E.; Urtti A.; del Amo E. M. Impact of Chemical Structure on Conjunctival Drug Permeability: Adopting Porcine Conjunctiva and Cassette Dosing for Construction of In Silico Model. J. Pharm. Sci. 2017, 106, 2463–2471. 10.1016/j.xphs.2017.04.061. 28479360

[ref24] Ramsay E.; del Amo E. M.; Toropainen E.; Tengvall-Unadike U.; Ranta V.-P.; Urtti A.; Ruponen M. Corneal and conjunctival drug permeability: Systematic comparison and pharmacokinetic impact in the eye. Eur. J. Pharm. Sci. 2018, 119, 83–89. 10.1016/j.ejps.2018.03.034. 29625211

[ref25] Cheng Y.-H.; Hung K.-H.; Tsai T.-H.; Lee C.-J.; Ku R.-Y.; Chiu A. W.; Chiou S.-H.; Liu C. J. Sustained delivery of latanoprost by thermosensitive chitosan-gelatin-based hydrogel for controlling ocular hypertension. Acta Biomater. 2014, 10, 4360–4366. 10.1016/j.actbio.2014.05.031. 24914827

[ref26] Song Z.; Gong Y.; Liu H.; Ren Q.; Sun X. Glycyrrhizin could reduce ocular hypertension induced by triamcinolone acetonide in rabbits. Mol. Vis. 2011, 17, 2056–2064. 21850181 PMC3156820

[ref27] Potts A. M. The Reaction of Uveal Pigment in vitro with Polycyclic Compounds. Invest. Ophthalmol. 1964, 3, 405–416. 14203332

[ref28] Salminen L.; Urtti A.; Periviita L. Effect of ocular pigmentation on pilocarpine pharmacology in the rabbit eye. I. Drag distribution and metabolism. Int. J. Pharm. 1984, 18, 17–24. 10.1016/0378-5173(84)90103-0.

[ref29] Kim Y. C.; Hsueh H. T.; Shin M. D.; Berlinicke C. A.; Han H.; Anders N. M.; Hemingway A.; Leo K. T.; Chou R. T.; Kwon H.; Appell M. B.; Rai U.; Kolodziejski P.; Eberhart C.; Pitha I.; Zack D. J.; Hanes J.; Ensign L. M. A hypotonic gel-forming eye drop provides enhanced intraocular delivery of a kinase inhibitor with melanin-binding properties for sustained protection of retinal ganglion cells. Drug Delivery Transl. Res. 2022, 12, 826–837. 10.1007/s13346-021-00987-6. PMC8546022 33900546

[ref30] Bahrpeyma S.; Rimpelä A.; Hagström M.; Urtti A. Ocular melanin binding of drugs: in vitro binding studies. Acta Ophthalmol. 2019, 97,S263 . 10.1111/j.1755-3768.2019.5366.

[ref31] Hsueh H. T.; Chou R. T.; Rai U.; Liyanage W.; Kim Y. C.; Appell M. B.; Pejavar J.; Leo K. T.; Davison C.; Kolodziejski P.; Mozzer A.; Kwon H.; Sista M.; Anders N. M.; Hemingway A.; Rompicharla S. V. K.; Edwards M.; Pitha I.; Hanes J.; Cummings M. P.; Ensign L. M. Machine learning-driven multifunctional peptide engineering for sustained ocular drug delivery. Nat. Commun. 2023, 14, 2509 10.1038/s41467-023-38056-w. 37130851 PMC10154330

[ref32] Jakubiak P.; Reutlinger M.; Mattei P.; Schuler F.; Urtti A.; Alvarez-Sánchez R. Understanding Molecular Drivers of Melanin Binding To Support Rational Design of Small Molecule Ophthalmic Drugs. J. Med. Chem. 2018, 61, 10106–10115. 10.1021/acs.jmedchem.8b01281. 30398862

[ref33] Pelkonen L.; Tengvall-Unadike U.; Ruponen M.; Kidron H.; del Amo E. M.; Reinisalo M.; Urtti A. Melanin binding study of clinical drugs with cassette dosing and rapid equilibrium dialysis inserts. Eur. J. Pharm. Sci. 2017, 109, 162–168. 10.1016/j.ejps.2017.07.027. 28756205

[ref34] Menon I. A.; Wakeham D. C.; Persad S. D.; Avaria M.; Trope G. E.; Basu P. K. Quantitative determination of the melanin contents in ocular tissues from human blue and brown eyes. J. Ocul. Pharmacol. 1992, 8, 35–42. 10.1089/jop.1992.8.35. 1402293

[ref35] Shinno K.; Kurokawa K.; Kozai S.; Kawamura A.; Inada K.; Tokushige H. The Relationship of Brimonidine Concentration in Vitreous Body to the Free Concentration in Retina/Choroid Following Topical Administration in Pigmented Rabbits. Curr. Eye Res. 2017, 42, 748–753. 10.1080/02713683.2016.1238941. 27854122

[ref36] Kalinin S.; Valtari A.; Ruponen M.; Toropainen E.; Kovalenko A.; Nocentini A.; Gureev M.; Dar’in D.; Urtti A.; Supuran C. T.; Krasavin M. Highly hydrophilic 1,3-oxazol-5-yl benzenesulfonamide inhibitors of carbonic anhydrase II for reduction of glaucoma-related intraocular pressure. Bioorg. Med. Chem. 2019, 27, 115086 10.1016/j.bmc.2019.115086. 31515057

[ref37] Pilipenko I.; Korzhikov-Vlakh V.; Valtari A.; Anufrikov Y.; Kalinin S.; Ruponen M.; Krasavin M.; Urtti A.; Tennikova T. Mucoadhesive properties of nanogels based on stimuli-sensitive glycosaminoglycan-graft-pNIPAAm copolymers. Int. J. Biol. Macromol. 2021, 186, 864–872. 10.1016/j.ijbiomac.2021.07.070. 34274401

[ref38] Razali N.; Agarwal R.; Agarwal P.; Kapitonova M. Y.; Kannan Kutty M.; Smirnov A.; Salmah Bakar N.; Ismail N. M. Anterior and posterior segment changes in rat eyes with chronic steroid administration and their responsiveness to antiglaucoma drugs. Eur. J. Pharmacol. 2015, 749, 73–80. 10.1016/j.ejphar.2014.11.029. 25481859

[ref39] Nagata A.; Mishima H.; Kiuchi Y.; Hirota A.; Kurokawa T.; Ishibashi S. Binding of antiglaucomatous drugs to synthetic melanin and their hypotensive effects on pigmented and nonpigmented rabbit eyes. Jpn. J. Ophthalmol. 1993, 37, 32–38. 8100592

[ref40] Salazar M.; Patil P. N. An explanation for the long duration of mydriatic effect of atropine in eye. Invest. Ophthalmol. Vis. Sci. 1976, 15, 671. 955835

[ref41] Park C. G.; Choi G.; Kim M. H.; Kim S.-N.; Lee H.; Lee N. K.; Choy Y. B.; Choy J.-H. Brimonidine-montmorillonite hybrid formulation for topical drug delivery to the eye. J. Mater. Chem. B 2020, 8, 7914–7920. 10.1039/d0tb01213k. 32726382

[ref42] Kim S.-N.; Ko S. A.; Park C. G.; Lee S. H.; Huh B. K.; Park Y. H.; Kim Y. K.; Ha A.; Park K. H.; Choy Y. B. Amino-Functionalized Mesoporous Silica Particles for Ocular Delivery of Brimonidine. Mol. Pharmaceutics 2018, 15, 3143–3152. 10.1021/acs.molpharmaceut.8b00215. 30020792

[ref43] Bhagav P.; Upadhyay H.; Chandran S. Brimonidine Tartrate-Eudragit Long-Acting Nanoparticles: Formulation, Optimization, In Vitro and In Vivo Evaluation. AAPS PharmSciTech 2011, 12, 1087–1101. 10.1208/s12249-011-9675-1. 21879393 PMC3225524

[ref44] Sharma P. K.; Chauhan M. K. Optimization and Characterization of Brimonidine Tartrate Nanoparticles-loaded In Situ Gel for the Treatment of Glaucoma. Curr. Eye Res. 2021, 46, 1703–1716. 10.1080/02713683.2021.1916037. 33844617

[ref45] Takahashi N.; Sato K.; Kiyota N.; Yamazaki M.; Kunikane E.; Nakazawa T. The effect of a brinzolamide/brimonidine fixed combination on optic nerve head blood flow in rabbits. PLoS One 2023, 18, e0295122 10.1371/journal.pone.0295122. 38051718 PMC10697578

[ref46] Soda M.; Yoshida M.; Onda H.; Guo S.-Y.; Nakanishi-Ueda T.; Ueda T.; Fujishiro N.; Hisamitsu T.; Inatomi M.; Yasuhara H.; Oguchi K.; Koide R. The Effect of Brimonidine on Intraocular Pressure via the Central Nervous System in the Conscious Pigmented Rabbit. Showa Univ. J. Med. Sci. 2003, 15, 313–322. 10.15369/sujms1989.15.313.

[ref47] Acheampong A. A.; Shackleton M.; Tang-Liu D. D.-S. Comparative ocular pharmacokinetics of brimonidine after a single dose application to the eyes of albino and pigmented rabbits. Drug Metab. Dispos. 1995, 23, 708–712. 7587958

[ref48] Bahrpeyma S.; Reinisalo M.; Hellinen L.; Auriola S.; del Amo E. M.; Urtti A. Mechanisms of cellular retention of melanin bound drugs: Experiments and computational modeling. J. Controlled Release 2022, 348, 760–770. 10.1016/j.jconrel.2022.05.059. 35738465

[ref49] Hsueh H. T.; Chou R. T.; Rai U.; Kolodziejski P.; Liyanage W.; Pejavar J.; Mozzer A.; Davison C.; Appell M. B.; Kim Y. C.; Leo K. T.; Kwon H.; Sista M.; Anders N. M.; Hemingway A.; Rompicharla S. V. K.; Pitha I.; Zack D. J.; Hanes J.; Cummings M. P.; Ensign L. M. Engineered peptide-drug conjugate provides sustained protection of retinal ganglion cells with topical administration in rats. J. Controlled Release 2023, 362, 371–380. 10.1016/j.jconrel.2023.08.058. PMC10591956 37657693

[ref50] SooHoo J. R.; Golas L.; Marando C. M.; Seibold L. K.; Pantcheva M. B.; Ramulu P. Y.; Kahook M. Y. Glaucoma Patient Treatment Preferences. Ophthalmology 2016, 123, 1621–1622. 10.1016/j.ophtha.2016.01.018. 26948306

[ref51] Varadaraj V.; Kahook M. Y.; Ramulu P. Y.; Pitha I. F. Patient Acceptance of Sustained Glaucoma Treatment Strategies. J. Glaucoma 2018, 27, 328–335. 10.1097/IJG.0000000000000913. 29462013

[ref52] Hedengran A.; Kolko M. The molecular aspect of anti-glaucomatous eye drops - are we harming our patients?. Mol. Aspects Med. 2023, 93, 101195 10.1016/j.mam.2023.101195. 37459821

[ref53] Newman-Casey P. A.; Robin A. L.; Blachley T.; Farris K.; Heisler M.; Resnicow K.; Lee P. P. The Most Common Barriers to Glaucoma Medication Adherence: A Cross-Sectional Survey. Ophthalmology 2015, 122, 1308–1316. 10.1016/j.ophtha.2015.03.026. 25912144 PMC4485580

[ref54] Feehan M.; Munger M. A.; Cooper D. K.; Hess K. T.; Durante R.; Jones G. J.; Montuoro J.; Morrison M. A.; Clegg D.; Crandall A. S.; DeAngelis M. M. Adherence to Glaucoma Medications Over 12 Months in Two US Community Pharmacy Chains. Jpn. Clin. Med. 2016, 5, 79 10.3390/jcm5090079. PMC5039482 27618115

[ref55] Nordstrom B. L.; Friedman D. S.; Mozaffari E.; Quigley H. A.; Walker A. M. Persistence and Adherence With Topical Glaucoma Therapy. Am. J. Ophthalmol. 2005, 140, 598.e1–598.e11. 10.1016/j.ajo.2005.04.051. 16226511

[ref56] Edelhauser H. F.; Maren T. H. Permeability of human cornea and sclera to sulfonamide carbonic anhydrase inhibitors. Arch. Ophthalmol. 1988, 106, 1110 10.1001/archopht.1988.01060140266039. 3401140

[ref57] Durairaj C.; Chastain J. E.; Kompella U. B. Intraocular distribution of melanin in human, monkey, rabbit, minipig and dog eyes. Exp. Eye Res. 2012, 98, 23–27. 10.1016/j.exer.2012.03.004. 22440812 PMC3358346

