# Supplementary material for: Melanin-Binding-Based Discovery of Topically Instilled Carbonic Anhydrase Inhibitors for Targeted Delivery and Prolonged Action in the Eye
Source: Mol Pharm. 2025 Jan 9;22(2):721–32. doi: 10.1021/acs.molpharmaceut.4c00694 (PMC11795524; doi:10.1021/acs.molpharmaceut.4c00694)
Supplement: Supplementary file 1 — mp4c00694_si_001.pdf [file mp4c00694_si_001.pdf]

Supplementary material

Melanin-binding-based discovery of topically instilled carbonic anhydrase inhibitors for targeted delivery and prolonged action in the eye

Valtari Annika[1], Kalinin Stanislav[1], Jäntti Janika[1], Vanhanen Pekka[1], Martina Hanzlikova[2], Arun Tonduru[1], Katja Stenberg[1], Tapani Viitala[2,3], Kati-Sisko Vellonen[1], Toropainen Elisa[1], Ruponen Marika[1], Urtti Arto[1,2*]

1. University of Eastern Finland, School of Pharmacy, Yliopistonranta 1 C, 70210 Kuopio, Finland
2. Drug Research Program, Faculty of Pharmacy, University of Helsinki, Viikinkaari 5 00014 Helsinki, Finland
3. Pharmaceutical Sciences Laboratory, Faculty of Science and Engineering, Åbo Akademi University, Tykistökatu 6A 20520 Turku, Finland

*Corresponding author:

Prof. Arto Urtti, University of Eastern Finland, School of Pharmacy, Yliopistonranta 1 C, 70210 Kuopio, Finland Tel: +358 40 355 3061; email:

arto.urtti@uef.fi

Appendix table 1. Molecular structures and descriptors of new CAI molecules. Molecular descriptors were obtained with ACDLabs 12.0 or from PubChem 2021.

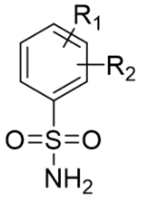

A01-A30

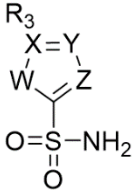

B01-B11

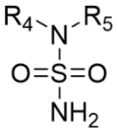

C01-C22

CAI	Molecular structure	Molecular descriptors						Melanin binding affinity			K_i or K_d against *h*CA II (nM)
		MW (g/mol)	$logD_{7.4}$	PSA (Å^2)	HB_{tot}	HB_d	Halogen ratio	Detector	Method of the analysis*	K_d ± confidence** (µM)	
A01	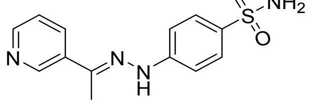	290.34	0.99	105.82	11	3	0	Nano	IF	48 ± 13	36.9 ± 23.4****
A02	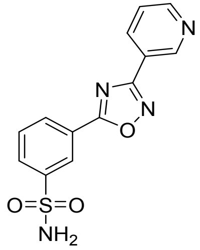	302.31	0.89	120.35	9	2	0	Nano	IF	78 ± 24	0.77 ± 0.08***
A03	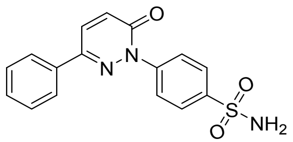	327.36	0.75	101.21	8	2	0	Nano	IF	78 ± 39	0.26 ± 0.03***

CAI	Molecular structure	Molecular descriptors						Melanin binding affinity			K_i or K_d against *h*CA II (nM)
		MW (g/mol)	$logD_{7.4}$	PSA ($Å^2$)	HB_{tot}	HB_d	Halogen ratio	Detector	Method of the analysis*	K_d ± confidence** (µM)	
A04	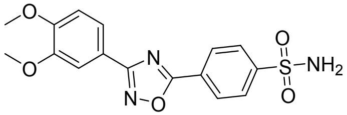	361.37	1.69	125.92	10	2	0.36	Nano	IF	92 ± 17	0.46 ± 0.05***
A05	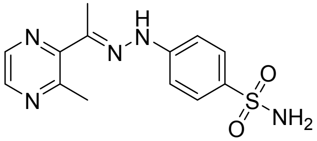	305.36	0.99	118.71	10	3	0	Nano	IF	97 ± 44	31.5 ± 9.2****
A06	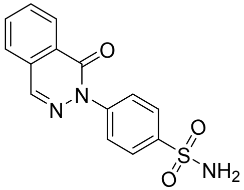	301.32	0.83	101.21	8	2	0	Nano	IF	104 ± 42	0.23 ± 0.02***
A07	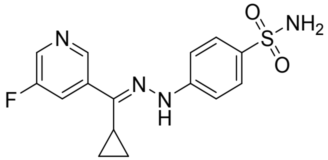	334.37	1.7	105.82	10	3	0.04	Nano	IF	115 ± 85	75.6 ± 11.2****
A08	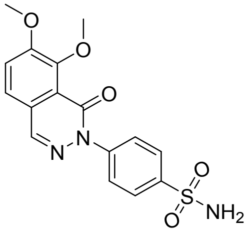	361.37	0.67	119.67	10	2	0	Nano	IF	142 ± 55	3.5 ± 0.4***
A09	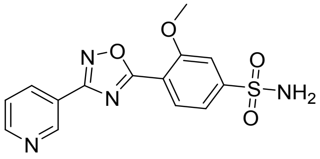	332.33	0.69	129.58	10	2	0	Nano	IF	152 ± 98	0.74 ±0.08***

CAI	Molecular structure	Molecular descriptors						Melanin binding affinity			K_i or K_d against *h*CA II (nM)
		MW (g/mol)	$logD_{7.4}$	PSA (Å^2)	HB_{tot}	HB_d	Halogen ratio	Detector	Method of the analysis*	K_d ± confidence** (µM)	
A10	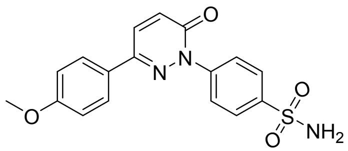	357.38	0.93	110.44	9	2	0	Nano	IF	156 ± 101	0.08 ± 0.001***
A11	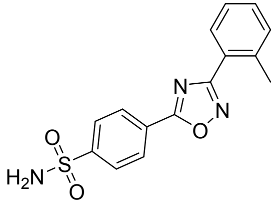	315.35	2.73	107.46	8	2	0	Nano	IF	177 ± 56	0.42 ± 0.04***
A12	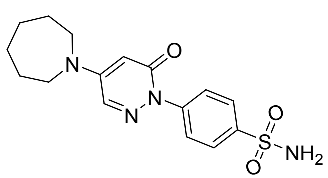	348.42	0.53	104.45	9	2	0	Nano	IF	176 ± 159	0.06 ± 0.001***
A13	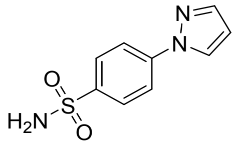	223.25	0.80	86.36	7	2	0	Nano	IF	203 ± 367	0.072 ± 0.001***
A14	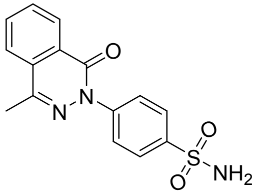	315.35	1.18	101.21	8	2	0	Nano	IF	205 ± 98	0.03 ± 0.01***
A15	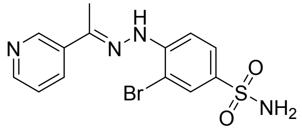	369.24	1.53	105.82	9	3	0.05	Nano	IF	230 ± 85	46.0 ± 4.6****

CAI	Molecular structure	Molecular descriptors						Melanin binding affinity			K_i or K_d against *h*CA II (nM)
		MW (g/mol)	$logD_{7.4}$	PSA ($Å^2$)	HB_{tot}	HB_d	Halogen ratio	Detector	Method of the analysis*	K_d ± confidence** (µM)	
A16	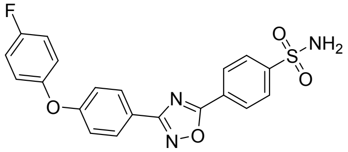	411.41	4.39	116.69	9	2	0	Nano	IF	451 ± 581	48.3 ± 4.8***
A17	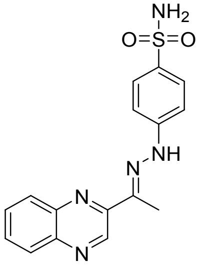	341.39	2.4	118.71	10	3	0	Nano	IF	483 ± 137	113.3 ± 11.3****
A18	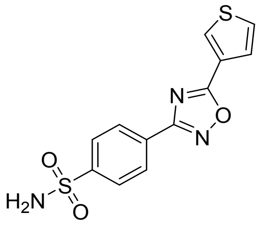	307.35	1.96	135.70	8	2	0	Nano	IF	520 ± 79	44.3 ± 4.4***
A19	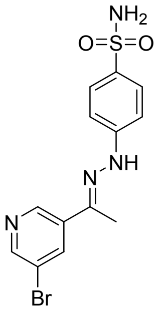	369.24	1.92	105.82	9	3	0.05	Nano	IF	561 ± 299	161.3 ± 16.1****

CAI	Molecular structure	Molecular descriptors						Melanin binding affinity			K_i or K_d against *h*CA II (nM)
		MW (g/mol)	$logD_{7.4}$	PSA (Å^2)	HB_{tot}	HB_d	Halogen ratio	Detector	Method of the analysis*	K_d ± confidence** (µM)	
A20	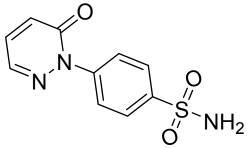	101.21	-1.37	101.21	8	2	0	Nano	IF	604 ± 3 690	8.2 ± 0.8***
A21	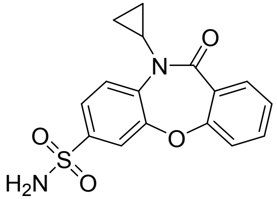	330.36	2.47	98.08	8	2	0	Nano	IF	722 ± 1033	0.82 ± 0.08***
A22	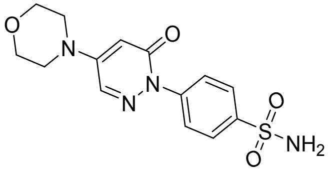	336.37	-1.43	113.37	10	2	0	Nano	IF	933 ± 1 450	0.55 ± 0.01***
A23	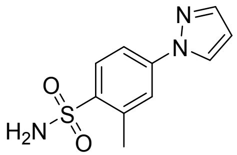	237.28	1.44	86.36	7	2	0	Nano	IF	957 ± 2010	0.008 ± 0.001***
A24	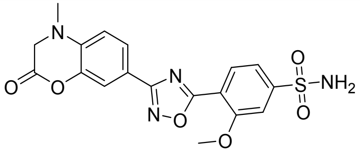	416.41	1.22	146.23	12	2	0	Nano	IF	1550 ± 4580	48.3 ± 4.8***
A25	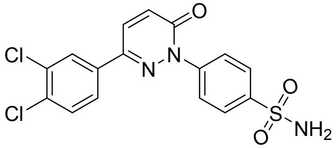	396.25	1.74	101.21	8	2	0.08	Nano	IF	6 700 ± 158 000	74.1 ± 7.4***

CAI	Molecular structure	Molecular descriptors						Melanin binding affinity			K_i or K_d against *h*CA II (nM)
		MW (g/mol)	$logD_{7.4}$	PSA (Å^2)	HB_{tot}	HB_d	Halogen ratio	Detector	Method of the analysis*	K_d ± confidence** (µM)	
A26	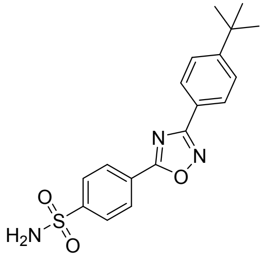	357.43	3.6	107.46	8	2	0	Nano	MST	13 200 ± 2.2×10^6	0.95 ± 0.09***
A27	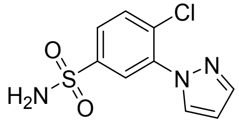	257.7	0.87	86.36	7	2	0.06	Nano	IF	31 240 ± 8720	0.001 ± 0.001***
A28	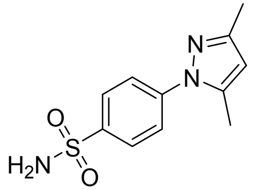	251.30	1.17	86.36	7	2	0	Nano	IF	38 500 ± 25 600	0.004 ± 0.001***
A29	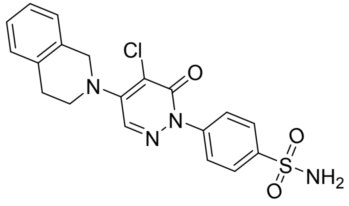	416.88	1.15	104.45	9	2	0.03	Nano	MST	50 000 ± 5×10^6	60.5 ± 6.0***
A30	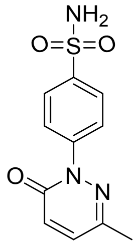	265.29	-0.7	101.21	8	2	0	Nano	-	Not detectable	0.68 ± 0.07***

CAI	Molecular structure	Molecular descriptors						Melanin binding affinity			K_i or K_d against *h*CA II (nM)
		MW (g/mol)	$logD_{7.4}$	PSA (Å^2)	HB_{tot}	HB_d	Halogen ratio	Detector	Method of the analysis*	K_d ± confidence** (µM)	
B01	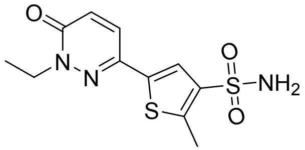	299.37	-0.29	129.45	8	2	0	Nano	IF	105 ± 30	4.8 ± 0.5***
B02	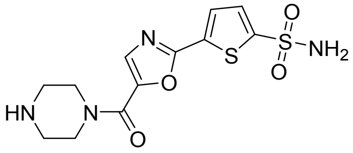	342.39	0.00	342.39	11	3	0	Nano	IF	165 ± 19	0.069 ± 0.007***
B03	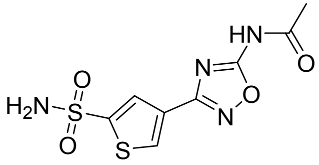	287.32	1.27	151.91	10	3	0	Nano	IF	231 ± 43	2.4 ± 0.2***
B04	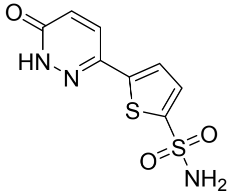	257.29	-0.29	138.24	9	3	0	Nano	IF	393 ± 185	3.6 ± 0.4***
B05	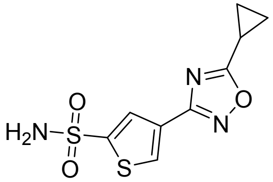	271.32	1.18	135.70	8	2	0	Nano	IF	448 ± 166	0.88 ± 0.08***
B06	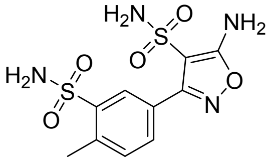	332.36	1.14	332.36	15	6	0	Nano	IF	573 ± 154	6.0 ± 0.6***

CAI	Molecular structure	Molecular descriptors						Melanin binding affinity			K_i or K_d against *h*CA II (nM)
		MW (g/mol)	$logD_{7.4}$	PSA (Å^2)	HB_{tot}	HB_d	Halogen ratio	Detector	Method of the analysis*	K_d ± confidence** (µM)	
B07	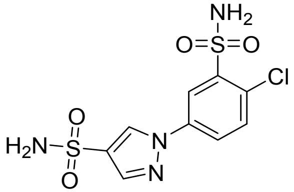	336.78	-1.65	154.90	12	4	0.05	Nano	IF	941 ± 1740	0.008 ± 0.001***
B08	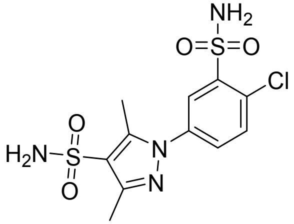	364.83	-1.09	154.90	12	4	0.05	Nano	IF	1 960 ± 13 500	0.008 ± 0.001***
B09	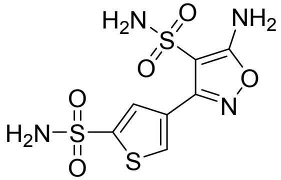	324.36	0.36	217.37	15	6	0	Nano	IF	2130 ± 7550	24.6 ± 2.5***
B10	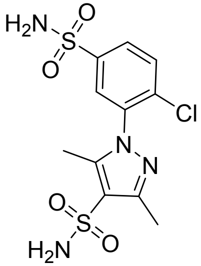	364.83	0.05	154.90	12	4	0.05	Nano	MST	50 000 ± 7.1×10^6	0.008 ± 0.01***
B11	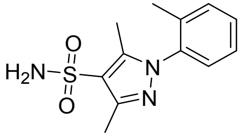	265.33	1.34	86.36	7	2	0	Nano	-	Not detectable	0.091 ± 0.01***

CAI	Molecular structure	Molecular descriptors						Melanin binding affinity			K_i or K_d against *h*CA II (nM)
		MW (g/mol)	$logD_{7.4}$	PSA ($Å^2$)	HB_{tot}	HB_d	Halogen ratio	Detector	Method of the analysis*	K_d ± confidence** (µM)	
C01	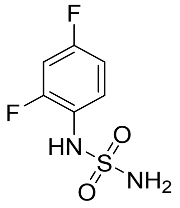	208.19	0.21	80.57	7	3	0.15	Pico	IF	92 ± 25	76.3 ± 7.6***
C02	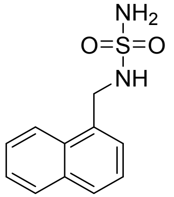	236.30	1.54	80.57	7	3	0	Pico	MST	122 ± 40	5250 ± 525***
C03	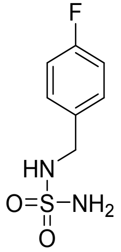	204.22	0.45	80.57	7	3	0.08	Pico	IF	361 ± 700	70.5 ± 7.1***
C04	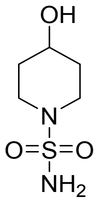	180.20	-1.5	92.01	8	3	0	Pico	IF	405 ± 311	42.7 ± 4.3***
C05	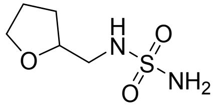	180.22	-1.11	89.8	8	3	0	Pico	IF	412 ± 802	63.4 ± 6.3***

CAI	Molecular structure	Molecular descriptors						Melanin binding affinity			K_i or K_d against *h*CA II (nM)
		MW (g/mol)	$logD_{7.4}$	PSA (Å^2)	HB_{tot}	HB_d	Halogen ratio	Detector	Method of the analysis*	K_d ± confidence** (µM)	
C06	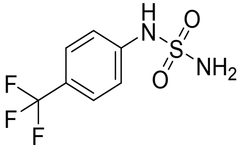	240.02	0.46	80.57	7	3	0.2	Pico	IF	582 ± 465	37.8 ± 3.8***
C07	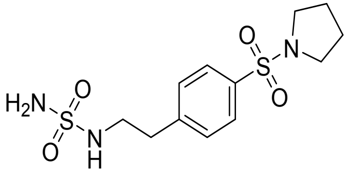	333.42	0.16	126.33	10	3	0	Pico	IF	596 ± 264	43.5 ± 4.4***
C08	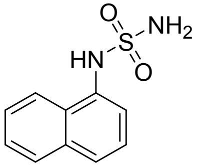	222.05	1.58	80.57	7	3	0	Pico	IF	1054 ± 2067	56.7 ± 5.7***
C09	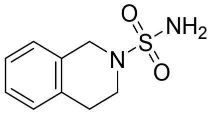	212.06	0.49	71.78	6	2	0	Pico	MST	1125 ± 2346	32.8 ± 3.3***
C10	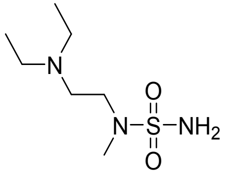	209.31	-2.08	75.02	7	2	0	Pico	MST	1522 ± 4032	92.3 ± 9.2***
C11	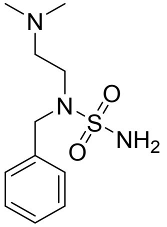	257.35	-0.82	75.02	7	2	0	Pico	MST	1625 ± 4531	39.8 ± 4.0***

CAI	Molecular structure	Molecular descriptors						Melanin binding affinity			K_i or K_d against *h*CA II (nM)
		MW (g/mol)	$logD_{7.4}$	PSA (Å^2)	HB_{tot}	HB_d	Halogen ratio	Detector	Method of the analysis*	K_d ± confidence** (µM)	
C12	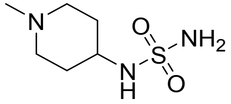	193.27	-1.52	83.81	8	3	0	Pico	IF	1675 ± 3476	52.1 ± 5.2***
C13	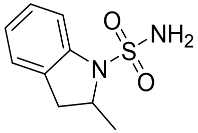	212.06	0.71	71.78	6	2	0	Pico	IF	1696 ± 6100	315.7 ± 31.6***
C14	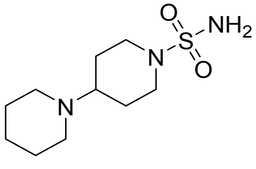	247.36	-1.38	75.02	7	2	0	Pico	IF	3576 ± 13 009	99.3 ± 9.9***
C15	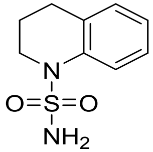	212.27	0.67	71.78	6	2	0	Pico	IF	8137 ± 96 580	89.5 ± 9.0***
C16	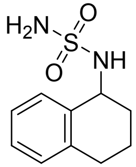	226.30	1.24	80.57	7	3	0	Pico	IF	25036 ± NA	42.6 ± 4.3***
C17	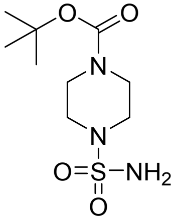	265.11	-0.14	101.32	9	2	0	Pico	-	Not detectable	16.2 ± 1.6***

CAI	Molecular structure	Molecular descriptors						Melanin binding affinity			K_i or K_d against *h*CA II (nM)
		MW (g/mol)	$logD_{7.4}$	PSA (Å[2])	HB_{tot}	HB_d	Halogen ratio	Detector	Method of the analysis*	K_d ± confidence** (µM)	
C18	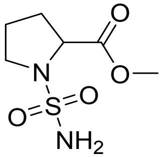	208.23	-0.95	98.08	8	2	0	Pico	-	Not detectable	69.8 ± 7.0***
C19	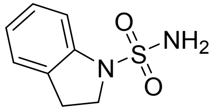	198.24	0.18	71.78	6	2	0	Pico	-	Not detectable	472.2 ± 47.2***
C20	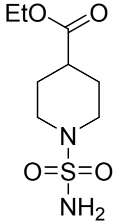	236.29	-0.23	98.08	8	2	0	Pico	-	Not detectable	42.8 ± 4.3***
C21	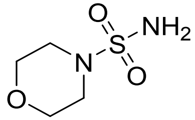	166.19	-1.57	81.01	7	2	0	Pico	-	Not detectable	66.9 ± 7.0***
C22	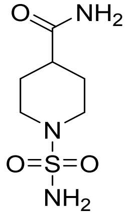	207.25	-1.77	114.87	6	2	0	Pico	-	Not detectable	59.4 ± 6.0***

CAI	Molecular structure	Molecular descriptors						Melanin binding affinity			K_i or K_d against *h*CA II (nM)
		MW (g/mol)	$logD_{7.4}$	PSA ($Å^2$)	HB_{tot}	HB_d	Halogen ratio	Detector	Method of the analysis*	K_d ± confidence** (µM)	
Dorzolamide	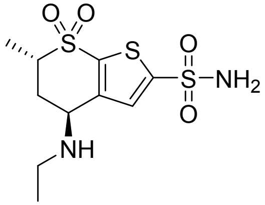	324.4	1.35	151.3	9	2	0	Nano	IF	431 ± 532	9.0 ± 0.9***<br>6.6±3.9****

* MST mode was utilized when K_d value could not be determined with initial fluorescence (IF) mode.
**The 68% confidence values for K_d were obtained within the MO. Affinity Analysis Software
NA = not available

*** K_i value measured against recombinant human carbonic anhydrase II via stopped flow kinetics protocol (references 12-19)

**** K_d value measured against recombinant human carbonic anhydrase II via surface plasmon resonance (Appendix Figs 5, 6)

Appendix table 2. Calculated permeability values of brinzolamide, dorzolamide and the experimental CAIs. The used quantitative structure property equations (QSPR) were published previously [1,2].

Compound	P_{app} in porcine cornea (cm/s)	P_{app} in porcine conjunctiva (cm/s)
Brinzolamide	1.64E-07	1.77E-06
Dorzolamide	1.75E-07	1.86E-06
**A01**	**2.96E-07**	**2.65E-06**
**A12**	**3.16E-07**	**2.80E-06**
**A22**	**2.96E-07**	**2.65E-06**
A02	2.84E-07	2.56E-06
A03	3.24E-07	2.85E-06
A04	2.74E-07	2.49E-06
A05	2.10E-07	2.15E-06
A06	3.24E-07	2.85E-06
A07	3.10E-07	3.21E-06
A08	2.85E-07	2.57E-06
A09	2.68E-07	2.45E-06
A10	3.03E-07	2.70E-06
A11	3.09E-07	2.75E-06
A13	3.66E-07	3.14E-06
A14	3.24E-07	2.85E-06
A15	3.17E-07	3.29E-06
A16	3.69E-07	3.39E-06
A17	2.10E-07	2.15E-06
A18	2.59E-07	2.38E-06
A19	3.17E-07	3.29E-06
A20	3.24E-07	2.85E-06
A21	3.32E-07	2.91E-06
A23	3.66E-07	3.14E-06
A24	2.44E-07	2.27E-06
A25	5.63E-07	5.23E-06
A26	3.09E-07	2.75E-06
A27	5.64E-07	5.04E-06
A28	3.66E-07	3.14E-06
A29	4.05E-07	3.66E-06
A30	3.24E-07	2.85E-06
B01	2.68E-07	2.45E-06
B02	1.71E-07	1.83E-06
B03	1.74E-07	1.85E-06
B04	1.87E-07	1.96E-06
B05	2.59E-07	2.38E-06
B06	5.81E-08	9.37E-07
B07	1.78E-07	2.23E-06
B08	1.72E-07	2.15E-06
B09	5.21E-08	8.61E-07
B10	1.72E-07	2.15E-06
B11	3.66E-07	3.14E-06

Compound	P_{app} in porcine cornea (cm/s)	P_{app} in porcine conjunctiva (cm/s)
C01	7.98E-07	8.51E-06
C02	2.83E-07	2.73E-06
C03	4.82E-07	4.89E-06
C04	2.52E-06	2.56E-07
C05	2.60E-07	2.56E-06
C06	1.13E-06	1.24E-05
C07	2.00E-07	2.07E-06
C08	2.83E-07	2.73E-06
C09	4.22E-07	3.52E-06
C10	4.08E-07	3.42E-06
C11	4.08E-07	3.42E-06
C12	2.75E-07	2.67E-06
C13	4.22E-07	3.52E-06
C14	4.08E-07	3.42E-06
C15	4.22E-07	3.52E-06
C16	2.83E-07	2.73E-06
C17	3.24E-07	2.85E-06
C18	1.39E-05	1.74E-04
C19	4.22E-07	3.52E-06
C20	3.32E-07	2.91E-06
C21	3.84E-07	3.27E-06
C22	2.94E-07	2.64E-06

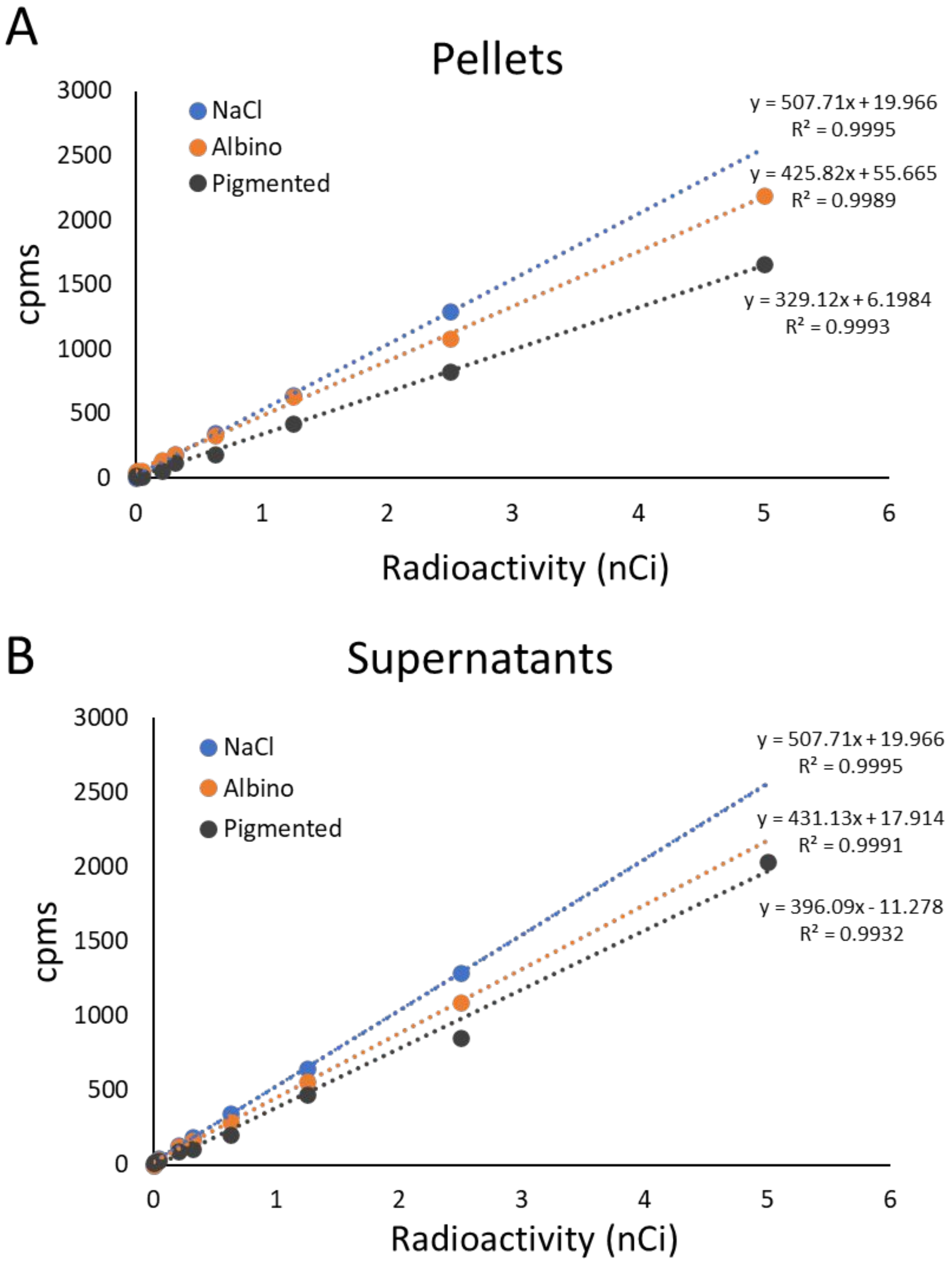

Appendix figure 1. Standard curves for quantitation of tritiated **A01** in tissue pellets (A) and supernatants (B) of rabbit iris-ciliary body. Standards in saline solution (0.9% NaCl) is also shown. Standard curves show higher colour quenching effect of the pigmented tissue samples.

Appendix Table 3. Signal to noise ratios of measured pellet and supernatant samples in pharmacokinetic rat study (n=4/each timepoint). Timepoints where 1-2 of measured values were very low (<0.015 ng/mg) are marked with orange color, and timepoints where 3-4 of measured values were very low (<0.015 ng/mg) are marked with red color.

Albino tissues

	**0.5**	**2**	**4**	**7**	**h**
**Pellet**	1.7–10.7	1.1–36.7	0.3–0.7	0.1–1.1	
**Supernatant**	14.5–100	9.3–14.1	1.8–7.2	1.9–6.0	

Pigmented tissues

	**0.5**	**2**	**4**	**7**	**24**	**48**	**72**	**144**	**240**	**h**
**Pellet**	65–94	128–391	147–241	122–412	121–295	90–162	31–73	16–76	2.6–44	
**Supernatant**	48–140	48–105	67–163	52–123	59–94	26–78	8.3–99	0.8–6.8	0.3–3.7	

Appendix equations 1-2. In silico predicted corneal and conjunctival permeability.

Equation 1. Predicted apparent corneal permeability (P_{app}) in porcine eye [2]:

$$LogPapp = -4.6823 - 0.7670 \times (logPSA) - 0.1346 \times (HBd) + 3.0024(Halogen\ ratio)$$

Equation 2. Predicted apparent conjunctival permeability (P_{app}) in porcine eye [1]:

$$LogPapp = -4.1594 - 0.6121 \times (logPSA) - 0.0792 \times (HBd) + 3.2914 \times (Halogen\ ratio)$$

In the equations, HB_{tot} is the total number of hydrogen bond forming moieties, $LogD_{7.4}$ the logarithm of distribution coefficient (n-octanol/water) at pH 7.4, LogPSA the logarithm of the polar surface area of the compound, HBd the number of hydrogen bond donors and Halogen ratio is the sum of all halogen atoms divided by the sum of all heavy atoms (i.e. hydrogens excluded). The chemical descriptors were determined for each molecule with ACDLabs software (version 12, Advanced Chemistry Development, Inc., Toronto, Canada).

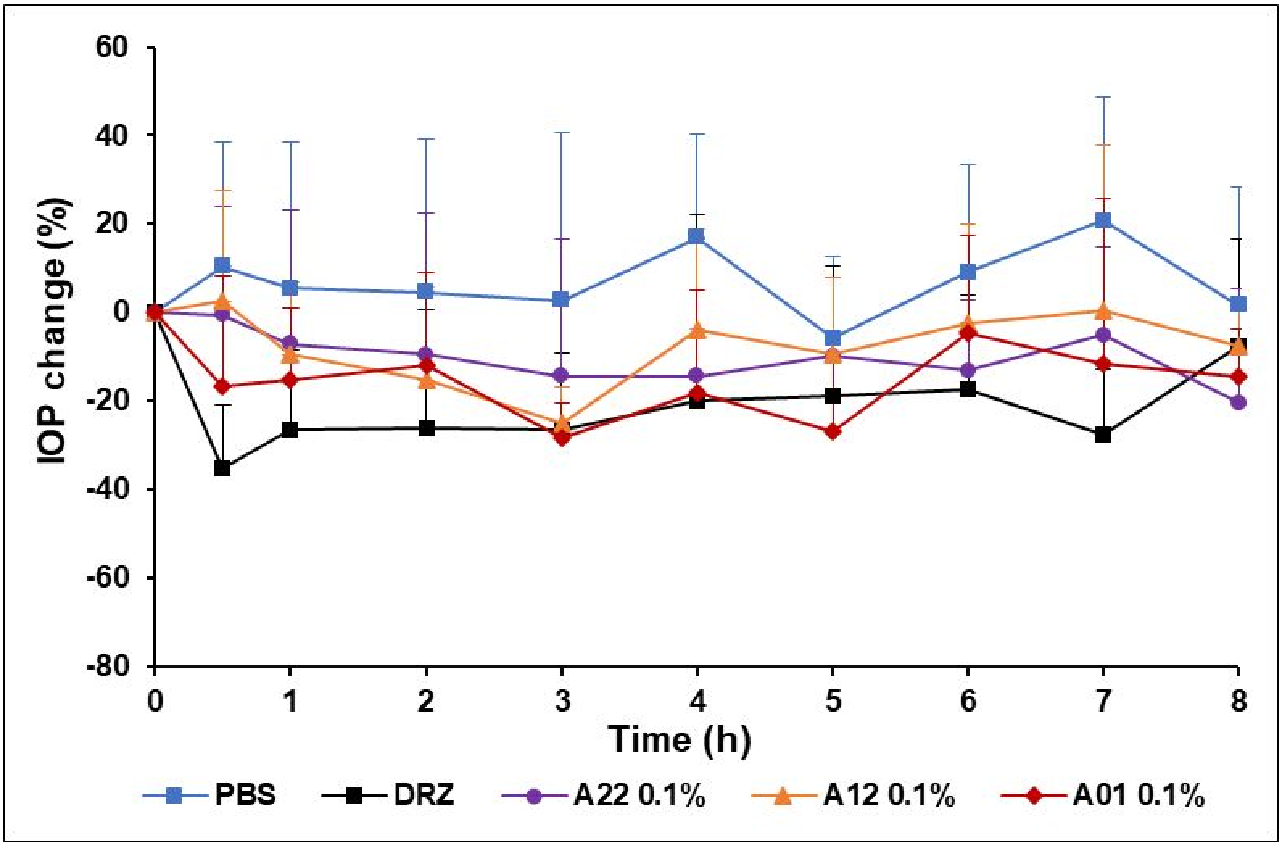

Appendix figure 2. The IOP-lowering effect (IOP decrease percentage ± SD) of low (**A22**), intermediate (**A12**), and high (**A01**) melanin binding CAI molecules given as 0.1% topical eye drops as compared to 2% dorzolamide (Trusopt®) and phosphate buffered saline (PBS) in albino rabbits (n=5 rabbits, one eye treated).

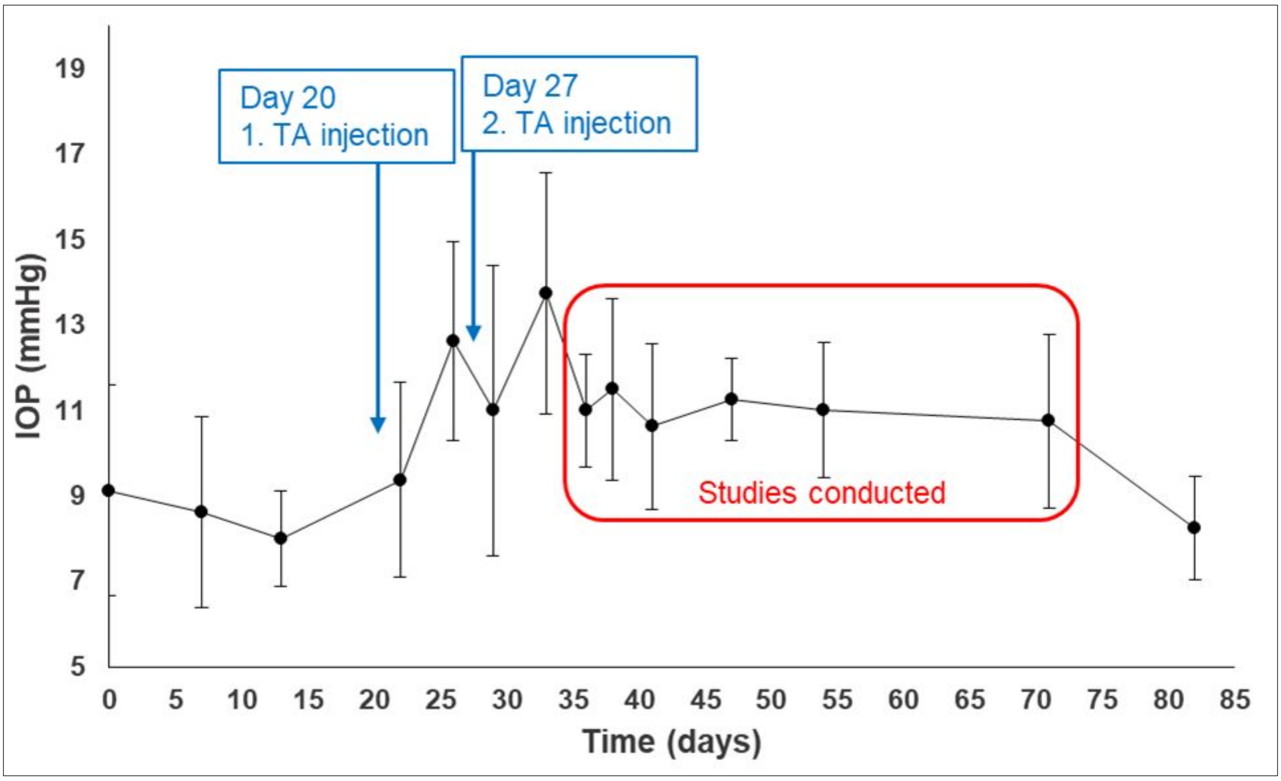

Appendix figure 3. Intraocular pressure (mmHg ± SD) before and after two intravitreal triamcinolone acetonide (TA) injections (method from previous studies [3,4]) to pigmented rabbits (n=5). The IOP baseline was followed 20 days before the first injection. After the second injection IOP was allowed to settle for 10 days before the experiments were performed. The IOP remained on constant hypertensive level for about 35 days before it started to decrease towards normal levels.

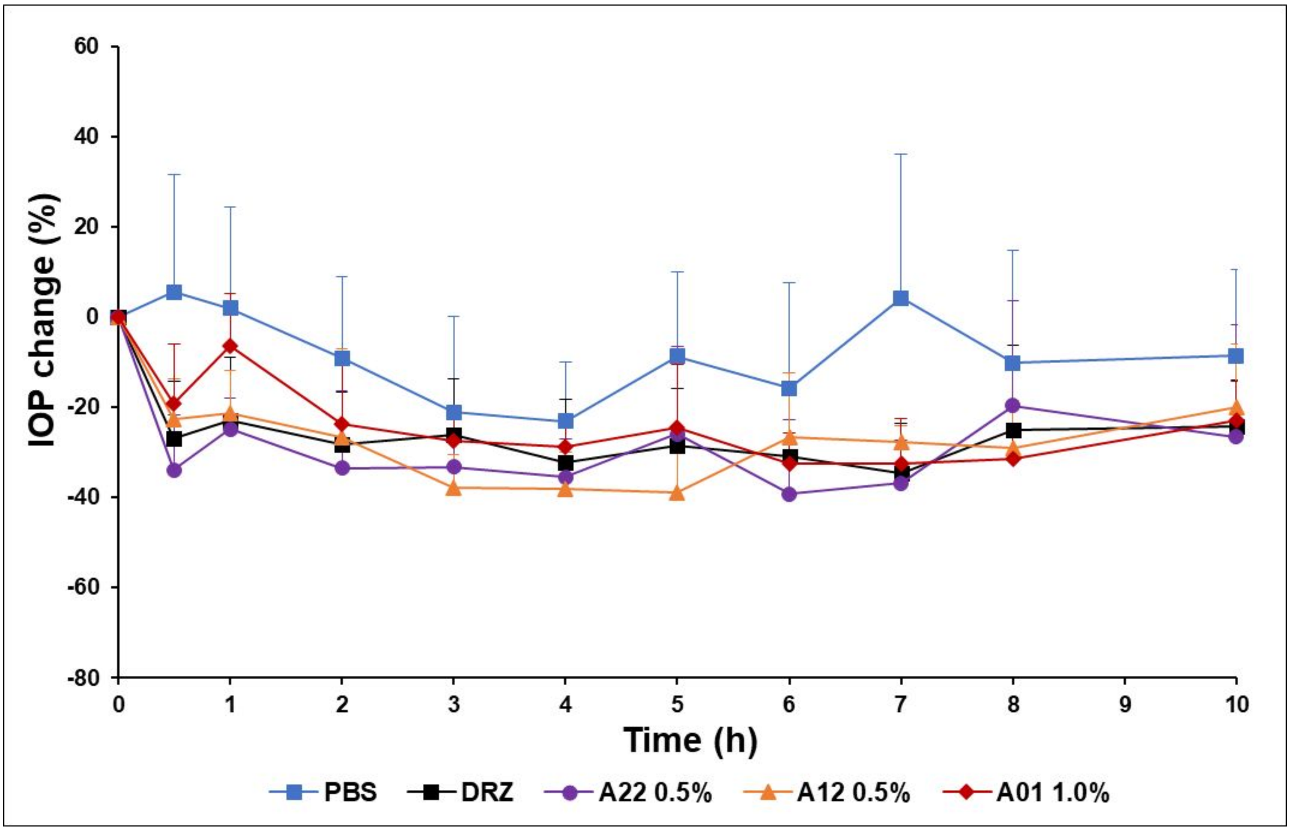

Appendix figure 4. IOP-lowering effect (IOP decrease as percentage ± SD) of new carbonic anhydrase inhibitors in pigmented rabbits (n=5) during 10 hours. PBS=Phosphate buffered saline, DRZ=dorzolamide, **A22**=CAI with low melanin binding, **A12**= CAI with intermediate melanin binding and **A01**= CAI with high melanin binding.

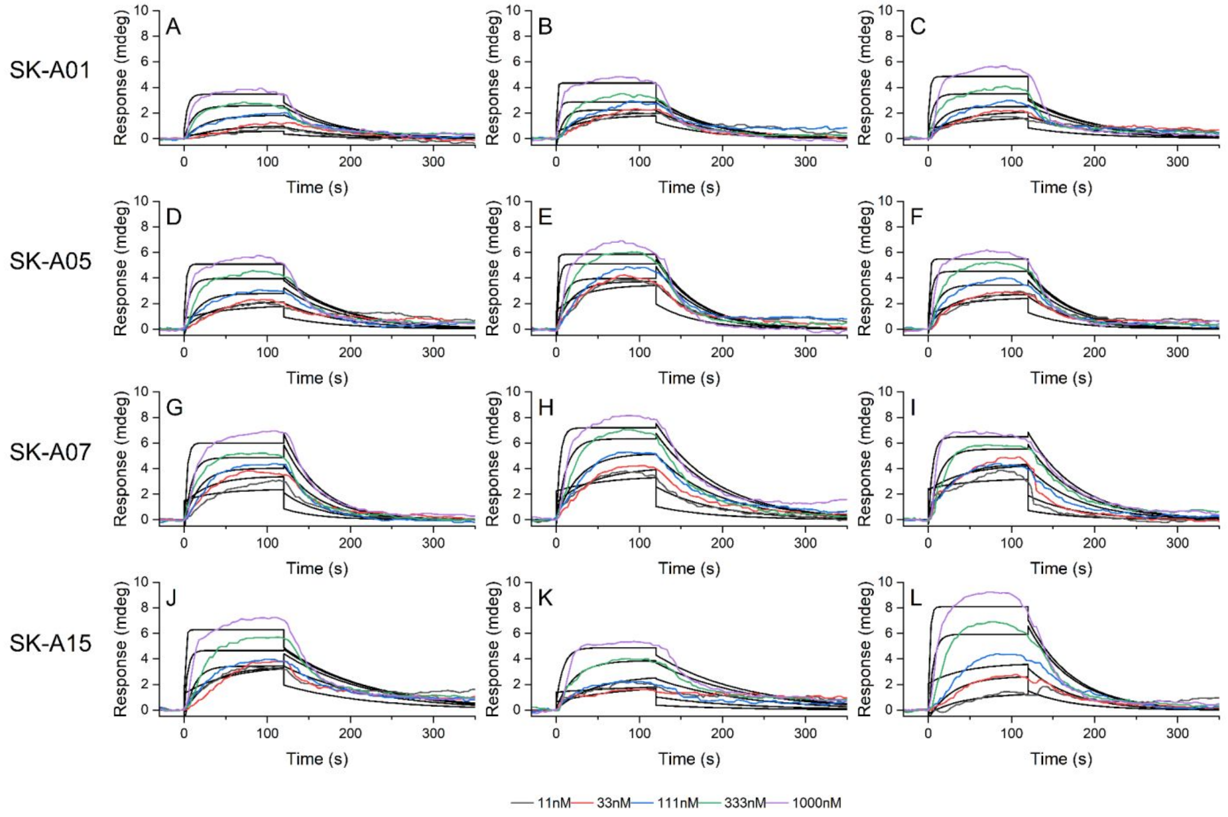

Appendix Fig. 5. Binding analysis of the interaction between various carbonic anhydrase inhibitors (CAIs) and human carbonic anhydrase II (hCAII) using multi-parametric surface plasmon resonance (MP-SPR). For all A01–A15 (SK-A01-SK-A15) the concentration range was 0 – 1μM. Coloured lines indicate MP-SPR signal responses, while black lines represent 1:1 model fits. Each row of the panel shows replicates.

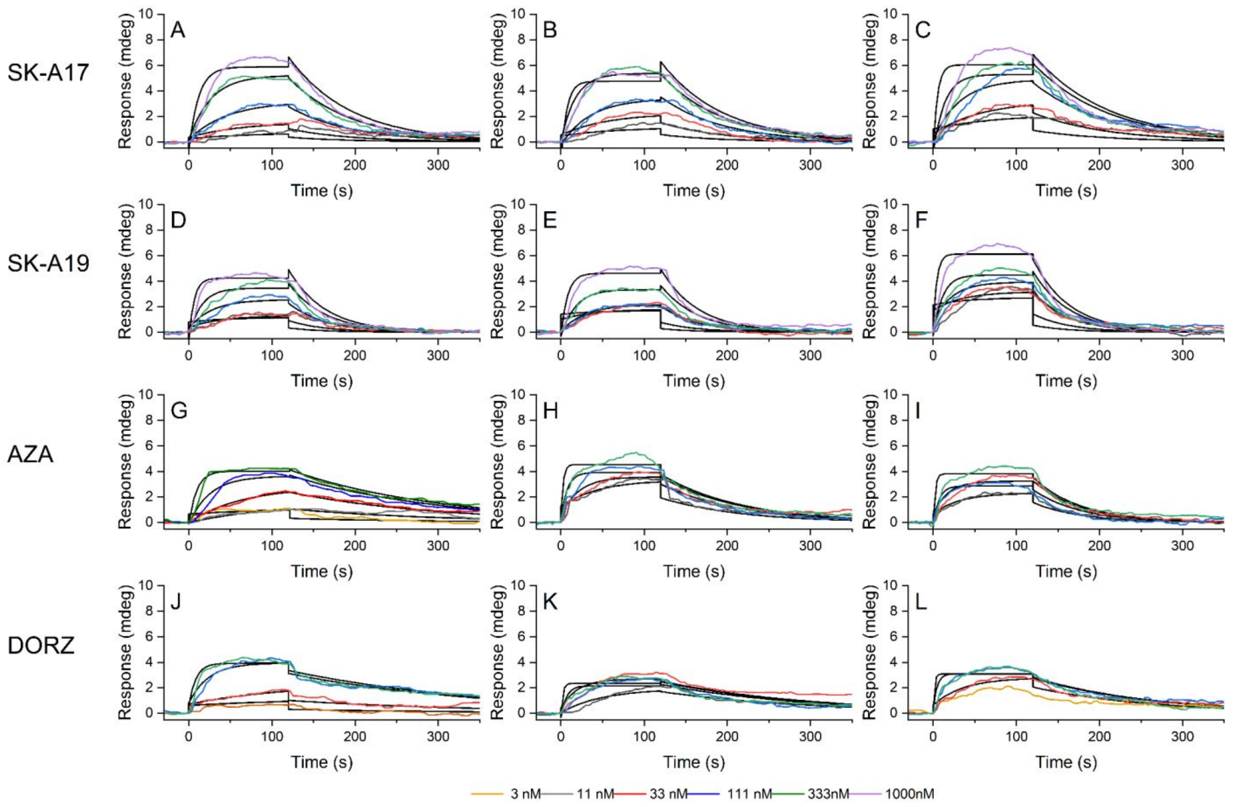

Appendix Figure 6. Binding analysis of the interaction between various carbonic anhydrase inhibitors (CAIs) and human carbonic anhydrase II (hCAII) using multi-parametric surface plasmon resonance (MP-SPR). For A17 (SK-A17) and A19 (SK-A19) the concentration range was 0 – 1µM, while for AZA and DORZ 0 – 333nM. Coloured lines indicate MP-SPR signal responses, while black lines represent 1:1 model fits. Each row of the panel shows replicates.

Supplementary material references:

[1] E. Ramsay, M. Ruponen, T. Picardat, U. Tengvall, M. Tuomainen, S. Auriola, E. Toropainen, A. Urtti, E.M. del Amo, Impact of Chemical Structure on Conjunctival Drug Permeability: Adopting Porcine Conjunctiva and Cassette Dosing for Construction of In Silico Model, J Pharm Sci 106 (2017) 2463–2471. https://doi.org/10.1016/j.xphs.2017.04.061.

[2] E. Ramsay, E.M. del Amo, E. Toropainen, U. Tengvall-Unadike, V.-P. Ranta, A. Urtti, M. Ruponen, Corneal and conjunctival drug permeability: Systematic comparison and pharmacokinetic impact in the eye, European Journal of Pharmaceutical Sciences 119 (2018) 83–89. https://doi.org/10.1016/j.ejps.2018.03.034.

[3] Y.-H. Cheng, K.-H. Hung, T.-H. Tsai, C.-J. Lee, R.-Y. Ku, A.W. Chiu, S.-H. Chiou, C.J. Liu, Sustained delivery of latanoprost by thermosensitive chitosan–gelatin-based hydrogel for controlling ocular hypertension, Acta Biomaterialia; Acta Biomater 10 (2014) 4360–4366. https://doi.org/10.1016/j.actbio.2014.05.031.

[4] Z. Song, Y. Gong, H. Liu, Q. Ren, X. Sun, Glycyrrhizin could reduce ocular hypertension induced by triamcinolone acetonide in rabbits, Mol Vis 17 (2011) 2056–2064.

From the reference list of the main text:

[12] T. Sharonova, P. Zhmurov, S. Kalinin, A. Nocentini, A. Angeli, M. Ferraroni, M. Korsakov, C.T. Supuran, M. Krasavin, Diversely substituted sulfamides for fragment-based drug discovery of carbonic anhydrase inhibitors: synthesis and inhibitory profile, J Enzyme Inhib Med Chem 37 (2022) 857–865. https://doi.org/10.1080/14756366.2022.2051023.

[13] M. Krasavin, A. Shetnev, S. Baykov, S. Kalinin, A. Nocentini, V. Sharoyko, G. Poli, T. Tuccinardi, M. Korsakov, T.B. Tennikova, C.T. Supuran, Pyridazinone-substituted benzenesulfonamides display potent inhibition of membrane-bound human carbonic anhydrase IX and promising antiproliferative activity against cancer cell lines, Eur J Med Chem 168 (2019) 301–314. https://doi.org/10.1016/j.ejmech.2019.02.044.

[14] M. Krasavin, A. Shetnev, T. Sharonova, S. Baykov, S. Kalinin, A. Nocentini, V. Sharoyko, G. Poli, T. Tuccinardi, S. Presnukhina, T.B. Tennikova, C.T. Supuran, Continued exploration of 1,2,4-oxadiazole periphery for carbonic anhydrase-targeting primary arene sulfonamides: Discovery of subnanomolar inhibitors of membrane-bound hCA IX isoform that selectively kill cancer cells in hypoxic environment, Eur J Med Chem 164 (2019) 92–105. https://doi.org/10.1016/j.ejmech.2018.12.049.

[15] A. Sapegin, S. Kalinin, A. Angeli, C.T. Supuran, M. Krasavin, Unprotected primary sulfonamide group facilitates ring-forming cascade en route to polycyclic [1,4]oxazepine-based carbonic anhydrase inhibitors, Bioorg Chem 76 (2018) 140–146. https://doi.org/10.1016/j.bioorg.2017.11.014.

[16] M. Krasavin, A. Shetnev, T. Sharonova, S. Baykov, T. Tuccinardi, S. Kalinin, A. Angeli, C.T. Supuran, Heterocyclic periphery in the design of carbonic anhydrase inhibitors: 1,2,4-Oxadiazol-5-yl benzenesulfonamides as potent and selective inhibitors of cytosolic hCA II and membrane-bound hCA IX isoforms, Bioorg Chem 76 (2018) 88–97. https://doi.org/10.1016/j.bioorg.2017.10.005.

[17] M. Krasavin, M. Korsakov, O. Ronzhina, T. Tuccinardi, S. Kalinin, M. Tanç, C.T. Supuran, Primary mono- and bis-sulfonamides obtained via regiospecific sulfochlorination of N-arylpyrazoles: inhibition profile against a panel of human carbonic anhydrases, J Enzyme Inhib Med Chem 32 (2017) 920–934. https://doi.org/10.1080/14756366.2017.1344236.

[18] M. Krasavin, M. Korsakov, Z. Zvonaryova, E. Semyonychev, T. Tuccinardi, S. Kalinin, M. Tanç, C.T. Supuran, Human carbonic anhydrase inhibitory profile of mono- and bis-sulfonamides synthesized via a direct sulfochlorination of 3- and 4-(hetero)arylisoxazol-5-amine scaffolds, Bioorg Med Chem 25 (2017) 1914–1925. https://doi.org/10.1016/j.bmc.2017.02.018.

[19] S. Kalinin, A. Kovalenko, A. Valtari, A. Nocentini, M. Gureev, A. Urtti, M. Korsakov, C.T. Supuran, M. Krasavin, 5-(Sulfamoyl)thien-2-yl 1,3-oxazole inhibitors of carbonic anhydrase II with hydrophilic periphery, J Enzyme Inhib Med Chem 37 (2022) 1005–1011. https://doi.org/10.1080/14756366.2022.2056733.
